# Natural sulfur compounds in mental health and neurological disorders: insights from observational and intervention studies

**DOI:** 10.3389/fnut.2025.1534000

**Published:** 2025-04-09

**Authors:** Apeksha Rana, Ashutosh Katiyar, Alok Arun, Juan Negron Berrios, Gaurav Kumar

**Affiliations:** ^1^School of Life Sciences and Biotechnology, CSJM University, Kanpur, India; ^2^Institute of Sustainable Biotechnology, Inter American University of Puerto Rico, Barranquitas, PR, United States; ^3^Department of Biological Sciences, California State University, Turlock, CA, United States

**Keywords:** antioxidant effects, natural sulfur compounds, neuroinflammation, neurological disorders, oxidative stress, dietary sources and mental disorders

## Abstract

Over the years, the global disease burden of neurological disorders (NDs) and mental disorders (MDs) has significantly increased, making them one of the most critical concerns and challenges to human health. In pursuit of novel therapies against MD and ND, there has been a growing focus on nutrition and health. Dietary sulfur, primarily derived from various natural sources, plays a crucial role in numerous physiological processes, including brain function. This review offers an overview of the chemical composition of several natural sources of the sulfur-rich substances such as isothiocyanates, sulforaphane, glutathione, taurine, sulfated polysaccharides, allyl sulfides, and sulfur-containing amino acids, all of which have neuroprotective properties. A multitude of studies have documented that consuming foods that are high in sulfur enhances brain function by improving cognitive parameters and reduces the severity of neuropathology by exhibiting antioxidant and anti-inflammatory properties at the molecular level. In addition, the growing role of natural sulfur compounds in repairing endothelial dysfunction, compromising blood–brain barrier and improving cerebral blood flow, are documented here. Furthermore, this review covers the encouraging results of supplementing sulfur-rich diets in many animal models and clinical investigations, along with their molecular targets in MD, such as schizophrenia, depression, anxiety, bipolar disorder, and autism spectrum disorder, and ND, such as Alzheimer’s disease (AD), Parkinson’s disease (PD), Amyotrophic Lateral Sclerosis (ALS), and Multiple Sclerosis (MS). The prospects of natural sulfur compounds show great promise as they have potential applications in nutraceuticals, medicines, and functional foods to enhance brain function and prevent diseases. However, additional research is required to clarify the mechanisms by which it works, enhance its bioavailability, and evaluate its long-term safety for broad use.

## 1 Introduction

Sulfur is an indispensable element necessary for our body’s metabolism and preventing diseases. Sulfur compounds can exist in various forms due to the wide range of oxidation states of sulfur, which can vary from −2 to +6 (1). Sulfides, thiols, disulfides, thioesters, thioketones, thioureas (−2), sulfoxides and sulfonates (+4), and sulfones and sulfonamides (+6) exhibit various oxidation states, highlighting the versatile role of sulfur in biochemistry (2). Many biological applications extensively use sulfur-containing compounds; for example, organosulfur compounds form some vitamins (biotin and thiamine) and amino acids (cysteine, cystine, and methionine) (3). Numerous sulfur-containing bioactive substances, such as taurine, hydrogen sulfide, and glutathione (GSH), contribute significantly to the health of living things by preserving cellular redox equilibrium. The sulfur-containing amino acids, cysteine and methionine, essential for the synthesis of proteins, hormones, coenzymes, and enzymes, fulfill our nutritional requirements for sulfur. In nature, there are many different kinds of sulfur: free sulfur, inorganic sulfurides and sulfates, sulfur oxides, sulfurous gases, and organosulfur compounds found in plant tissues and living things (4). One of the central metabolic pathways linked to sulfur, GSH, oxidative stress, and hydrogen sulfide production is the transsulfuration pathway (TSP) (5). It plays a critical role in regulating sulfur equilibrium and ensuring optimal cellular processes, including the formation of GSH (6). Perturbation of these pathways is critical to many neurological disorders (NDs) such as Alzheimer’s disease (AD), Parkinson’s disease (PD), Multiple sclerosis (MS), Amyotrophic Lateral Sclerosis (ALS), and Cerebral ischemia, indicating their involvement in the pathogenesis and advancement of these conditions (7). Moreover, there is a growing interest in sulfur metabolism and mental disorders (MDs) such as schizophrenia, depression, anxiety, bipolar disorder, and autism spectrum disorder. It has drawn the interest of the scientific community in supplementation with transsulfuration intermediates to alleviate neurological conditions. Several studies have shown that natural sulfur interventions can improve the integrity of cell membranes, make neurons less vulnerable to damage, lower oxidative stress, reduce neuroinflammation caused by different pro-inflammatory cytokines, and protect cells against excitotoxicity caused by abnormal neurotransmitter release from neurons and astrocytes (8). In this context, understanding the mechanistic role of supplementation of natural sulfur compounds in various ND is of utmost interest.

Despite this, sulfur-rich compounds, such as Glutathione (GSH), sulforaphane (SFN), taurine, sulfated polysaccharides, allyl sulfides, and cysteine and methionine in the form of sulfur-containing secondary metabolites, provide an important yet often overlooked source of sulfur. Natural sulfur compounds are widely present in multiple plant- and animal-based foods. Some of the main plant sources of natural sulfur compounds include cauliflower, Brussels sprouts, and cabbage, while animal sources include meat, fish, chicken, and eggs (8). Because of their multiple pharmacological benefits, natural sulfur compounds are often taken as a supplement. However, their role in ND has recently drawn attention. Numerous health benefits are associated with high consumption of sulfur-rich compounds, including glycemic, neuroprotective, anti-inflammatory, and antioxidant effects (9). There are various natural sources of sulfur-rich compounds that have shown encouraging outcomes in various NDs in different animal model systems and clinical studies. This review sheds light on the importance of natural sulfur-containing compounds, their sources, and their impact on mental disorders and neurological illnesses. This article presents a comprehensive analysis of the physiological functions of sulfur and its compounds in the brain. Understanding the molecular mechanisms of sulfur dysregulation and its role in the development of neurodegenerative and neuropsychiatric illnesses could accelerate future research.

## 2 Search strategy

We performed an extensive literature review utilizing sources such as PubMed, Google Scholar, Scopus, and Web of Science to collect relevant articles on natural sulfur compounds and their significance in MD and ND. We searched keywords terminology such as: “Sulfur compounds” OR “Sulfur compounds and neurological disorders” OR “neuroprotection” OR “neurodegenerative diseases” OR “Alzheimer’s” OR “Parkinson’s” OR “glutathione” OR “taurine” OR “sulforaphane” OR “dietary sulfur” OR “mental disorder” AND “antioxidant properties.” We ensured that the search included peer-reviewed articles published within the last 20 years; however, significant earlier studies were considered if they had historical relevance. Both *in vivo* (animal models) and *in vitro* investigations, together with clinical trials, were taken into account. We emphasized research related to the neuroprotective, antioxidant, and anti-inflammatory properties of these substances. We excluded works of literature that do not specifically pertain to the molecular mechanisms or therapeutic advantages of sulfur compounds in neurological situations or that concentrate on unrelated disorder mechanisms.

## 3 Dietary requirements of sulfur

**Figure 1:**
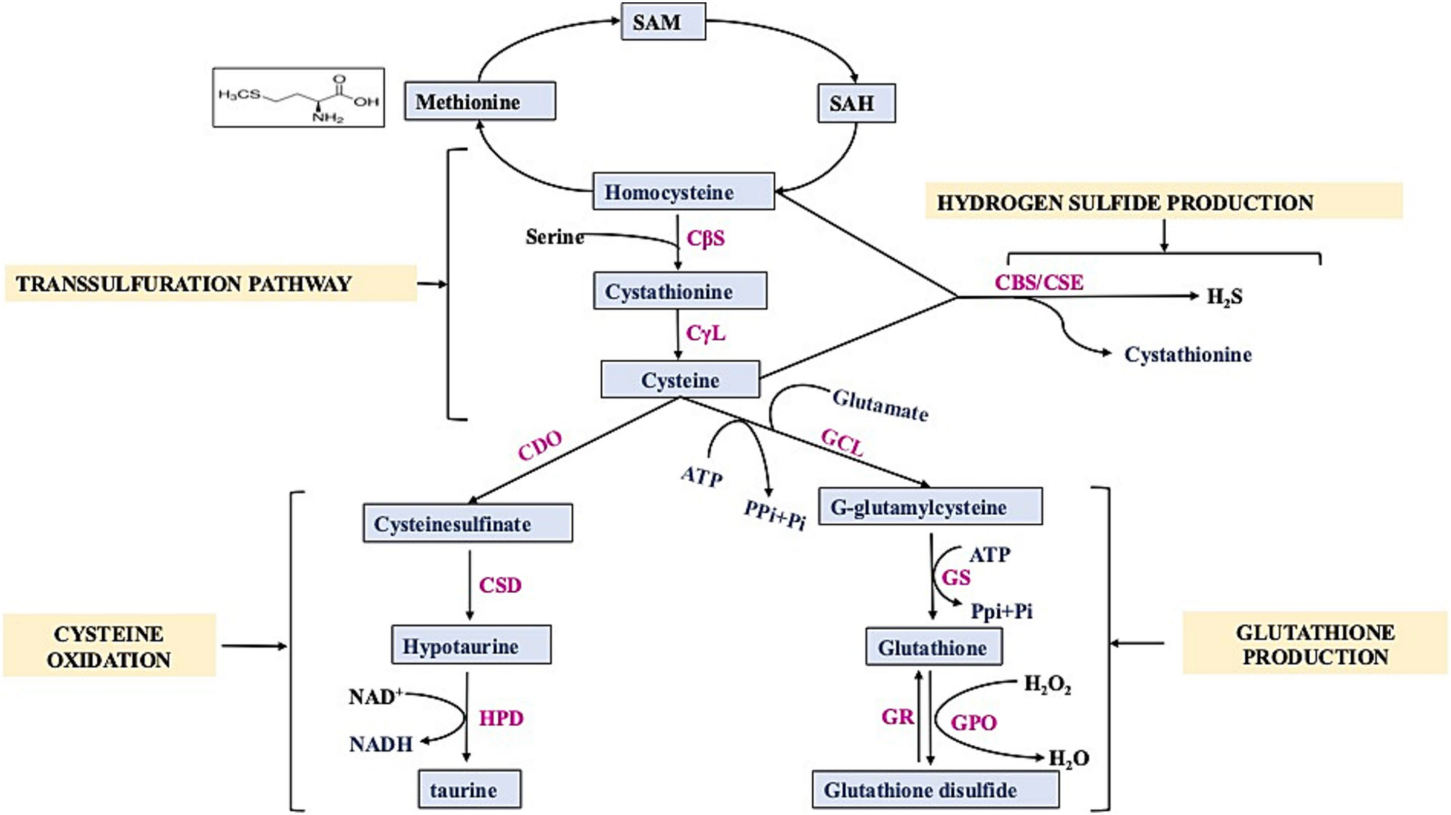
Schematic diagram showing transsulfuration pathway. SAM: S-Adenosyl- methionine, SAH: S-Adenosyl-homocysteine, CBS: Cystathionine β-Synthase, CSE/C*γ*L: Cystathionine γ-Lyase, CDO: Cysteine Dioxygenase, CSD: cysteine sulfinic acid decarboxylase, GCL: γ-glutamylcysteine synthetase, GS: glutathione synthetase, GR: glutathione reductase, GPO: Glutathione peroxidase, HPD: hypotaurine dehydrogenase.

Sulfur is used by our body for a number of critical processes; for instance, it works as an antioxidant and anti-inflammatory agent, aids in the neutralization of free radicals, and assists in preventing injury to the cells due to oxidative stress and various related issues (3). So, it is essential that the diet must contain enough items that are high in sulfur. At this time, there is no recommended daily consumption of sulfur; the only exception is for amino acids that contain sulfur. Humans require 13–15 mg/kg of sulfur-containing amino acids per day (and up to 89% of this can be supplied as cystine, the oxidized disulfide form of cysteine) (10). The determination of dietary sulfur requirements mostly relies on the assessment of the sulfur-containing amino acids, namely cysteine and methionine. Mammals can only get methionine through their diets (nuts, soy, and other beans); however, via the TSP, methionine can be converted into cysteine. The TSP is a crucial component of cellular sulfur metabolism and redox control (11). In mammals, the mechanism facilitates the transfer of sulfur from homocysteine to cysteine through cystathionine (Figure 1). This pathway is the exclusive means of biosynthesizing cysteine. Cystathionine synthase (CBS) converts homocysteine from dietary methionine to cystathionine, and cystathionine lyase (CSE) further processes this to produce cysteine (12). Besides sulfur amino acids, there are natural sulfur-containing compounds, e.g., GSH, SFN, taurine, sulfated polysaccharides, and allyl sulfides. The range and abundance of these sulfur-containing compounds are vast, and their impact on the human brain is significant. Some meals include the thiol-rich substance GSH, which promotes antioxidant defenses, principally through raising GSH levels and enzymes associated with glutathione’s action. Cysteine, glycine, and glutamic acid combine to form the tripeptide GSH (13). It is present in significant amounts in various body tissues. It has a crucial function in decreasing oxidative stress, preserving redox equilibrium, and improving metabolic detoxification. Researchers have linked age-related illnesses, such as neurodegeneration and mitochondrial dysfunction, to inadequate or insufficient amounts of GSH (14). The daily consumption of GSH varied between 13.0 and 109.9 mg, with an average of 34.8 mg (15). More than 50% of the typical dietary intake of GSH comes from fruits and vegetables, while less than 25% comes from meats (16). Cysteine, synthesized from homocysteine through the transsulfuration process, controls the production of GSH, the most prevalent antioxidant in mammals. Cruciferous vegetables naturally contain SFN, an isothiocyanate, as glucoraphanin. For a long time, people have recognized the medicinal characteristics of vegetables that contain high levels of sulfur-containing glucosinolates, which give rise to isothiocyanates. SFN, at a daily dosage ranging from 10 to 50 mg/kg, effectively avoided memory deterioration commonly associated with NDs (17). Nutritional and functional dietary isothiocyanates disrupt the molecular pathways involved in MD and ND development. Studies have demonstrated that the treatment of SFN may boost endogenous antioxidant enzymes and inflammatory indicators (18). An investigation was conducted to assess the intake of broccoli and the availability of SFN from various sources (19). Food provides a plentiful supply of taurine, a non-protein amino acid that the cysteine oxidation pathway also produces. Taurine, an endogenous amino acid that contains sulfur, has garnered much interest in recent times because of its potential advantages in promoting brain well-being (20). Dietary intake is necessary to obtain taurine, a crucial element for brain growth. Taurine levels in the human brain are typically reported to be approximately 1–2 mM, while in the mouse brain, they are approximately 5–10 times greater (21). It is present in various foods and is commonly utilized in energy drinks and supplements. Taurine is a significant byproduct of methionine metabolism in mammals. Cysteine, which can be derived from the sulfur of methionine through the TSP, seems to undergo conversion into taurine via the cysteine sulfinate pathway and serves as an osmolyte, a neuromodulator, an immunomodulator, and an antioxidant (22). Other natural compounds also modulate sulfur metabolism such as pterostilbene. Pan et al. performed transcriptomic profiling which demonstrated that Pterostilbene influenced the expression of genes related to sulfur metabolism by downregulating six genes (ECM17, MET3, MET14, MET16, MET10, and MET6) that encode enzyme regulating methionine biosynthesis (23). Recent studies have linked taurine to the stabilization of mitochondrial activity and intracellular pH buffering (24). Multiple pieces of evidence indicate the necessity of sulfur in our diet for numerous physiological functions that are crucial to human health. Therefore, it is imperative to evaluate guidelines for daily sulfur consumption.

## 4 Natural sources of sulfur-containing compounds

Sulfur is a vital component of all living organisms; it is present in two of the typical 20 amino acids found in proteins and can also be obtained primarily from diets. Abundant sources of natural sulfur compounds having physiological role include vitamins and amino acids containing sulfur compounds, such as methionine, cysteine, homocysteine, cystine, and taurine. Methionine is an essential amino acid that must be obtained from external sources as it cannot be synthesized physiologically. Cysteine is endogenously synthesized and can be supplemented as per the physiological requirement. Methionine and cysteine are linked to the transulfuration pathway, which includes significant intermediates such as taurine, homocysteine, and cystine (25). Methionine and cysteine are not stored by the body in their pure forms, but cysteine can be stored as GSH, another natural sulfur compound (26). GSH is present in a wide array of plant species, with its level varying across different plant species reported in Table 1. Eating fruits and vegetables high in polyphenols can increase the body’s production of GSH (16). Green peppers, apples, bananas, carrots, spinach, and cauliflower are among the fruits and vegetables that have a high concentration of GSH (27). Numerous herbs and roots, such as milk thistle, rosemary, turmeric/curcumin, and *ginkgo biloba*, may affect GSH levels, according to a number of animal studies (28). SFN is a member of the isothiocyanate family of compounds with antioxidant and anti-inflammatory properties. Plants in the genus Brassica are rich in glucoraphanin, which is hydrolyzed by the enzyme myrosinase to yield SFN, a secondary metabolite (29). Many cruciferous and Brassicaceae family plants, notably broccoli, brussel sprouts, and cabbage, contain high levels of sulfur-containing glucosinolates, specifically glucoraphanin, a precursor to SFN (30). SFN is enhanced in broccoli sprouts, which have a value of 1.153 mg/100 g, which is 20–50 times more concentrated than mature broccoli, and broccoli sprout extract contained 16.6 μmol of glucoraphanin per gram of fresh weight (31). Other dietary sources of SFN, along with their content values, are reported in Table 1.

**Table 1:** Concentration of sulfur compounds in different vegetables and fruits.

Vegetables and Fruits	Glutathione (μg/g)	Cysteine (μg/g)	Sulforaphane (μg/g)	Total polysulfide content (μg/g)
Arugula	NE	NE	110	NE
Asparagus	0.349	0.122	69.8	27.4
Avocado	0.339	0.004	NE	NE
Broccoli	0.004	NE	260	473.2
Carrot	0.004	NE	NE	51.4
Cabbage	NE	NE	10.1	101.8
Cauliflower	0.006	0.007	NE	NE
Cucumber	0.123	0.011	NE	111.4
Garlic	NE	NE	NE	314.1
Green beans	0.23	0.067	NE	NE
Green squash	0.047	0.006	NE	NE
Lettuce	NE	NE	NE	9.5
Mango	5.9	1	NE	NE
Onion	NE	NE	NE	782.8
Orange	0.5	4.1	NE	NE
Parsley	0.017	0.008	NE	NE
Spinach	0.313	0.084	NE	NE
Strawberry	3.9	5.9	NE	NE
Tomato	0.064	0.055	NE	5.04

NE: not evaluated. Concentrations are given in μg/g (micrograms per gram). References: Demirkol et al. (32), Minich et al. (28), and Kasamatsu et al. (33).

Garlic, onions, shallots, leeks, and chives are all members of the Allium genus and contain a variety of sulfur compounds; the sulfur content of onion is as high as 0.5–1% of dry weight (34). The Allium genus is also a rich source of allyl sulfides, which have recently gained attention as they are H_2_S donors with various physiological roles.

Taurine is a sulfonic amino acid that can be synthesized by other sulfonic amino acids, such as cysteine and methionine. However, the body cannot produce enough taurine on its own; food must be consumed to meet this need (35). It is estimated that adult non-vegetarians consume between 40 and 400 mg of taurine on a daily basis. Meat, fish, and dairy products from animals are good sources of taurine. It can be found in many animal products and byproducts, such as dairy, beef, dark meat poultry, and shellfish (36). Taurine is a popular ingredient in many energy drinks, and seafoods such as tuna, octopus, scallops, squid, white fish, small and medium prawns, mussels, oysters, cod, and clams are also high in taurine (37). Another natural source of sulfur compounds belongs to sulfated polysaccharides, which are complex carbohydrates having sulfate groups (-SO4) linked to their sugar molecules. These substances are predominantly found in marine species, such as algae, seaweed, and certain marine mammals, but they can also be found in terrestrial organisms. Agar, a kind of sulfated galactan, is an important sulfated polysaccharide (SP) utilized in the food processing sector (38). Seaweeds are plentiful sources of polysaccharides containing sulfate, which have several commercial applications in the culinary, cosmetic, and medicinal sectors (39). Seaweeds include sulfated polysaccharides with concentrations ranging from 4 to 76%. Majority of them consists of green (ulvans), red (carrageenan), and brown algae (fucoidan) (39). In subsequent sections, the molecular structure of natural sulfur compounds (e.g., GSH, SFN, taurine, sulfated polysaccharides, and allyl sulfides), their medicinal properties, and mechanistic insights in various ND are discussed.

## 5 Glutathione

GSH is a non-protein thiol crucial for maintaining the balance of cellular redox reactions. It is present in mammalian cells in varying amounts, typically ranging from 0.5 to 10 mM, depending on the specific tissues (40). GSH is a compound made up of three amino acids: gamma-glutamyl-cysteinyl-glycine. Gamma-glutamyl-cysteine is produced when the amino acids L-glutamate combine with L-cysteine in the presence of the enzyme glutamate-cysteine ligase, and this rate-limiting step is ATP-dependent. Subsequently, glycine is attached to *γ*-glutamyl cysteine, which is catalyzed by GSH synthase to synthesize GSH (41). GSH is synthesized solely in the cytosol and actively transported into mitochondria. It is present in cells in two forms: reduced, known as GSH, and oxidized as GSSG (glutathione disulfide) (42). Oxidized GSH consists of two reduced glutathione that are chemically linked together at the sulfur atoms. The ratio of GSH to GSSG is determined by the cellular redox state (42).

### 5.1 Applications of GSH in animal models of mental and neurological disorders

MD and ND have been closely associated with elevated oxidative stress and decreased GSH levels (43–48) (Table 2). Research indicates that GSH levels were found to be reduced in regions, i.e., hippocampus, substantia nigra, and frontal cortex, which are susceptible to AD and PD and contribute to neuronal cell death in ALS (49). A study has been conducted on the GCLM knockout mice (GCLM-KO) model of schizophrenia to investigate the impact of brain GSH level on myelination in the prefrontal cortex, which suggests that its deficiency affects oligodendrocyte maturation and myelination (50). Neuronal-astrocyte metabolic association is essential for GSH synthesis, and it has been conducted on co-cultured astrocytes and neurons in which the presence of astrocytes leads to elevated GSH levels in neurons. This increase is most probably due to the transport of cysteine precursor from astrocytes to neurons, which in turn enhances the formation of GSH in the recipient neurons (51).

**Table 2:** Overview of sulfur-containing compounds, their sources, and observed effects in experimental models of mental and neurological disorders.

Sulfur-containing compound	Source	Model/system	Disorders	Significant findings	References
Glutathione	Meat, dairy products, fruits and vegetables (asparagus, avocado, Spinach)	Adult male Sprague–Dawley rats and C57Bl/6 mice	Alzheimer’s	Improved cognitive decline and depressive-like behavior	(44)
		SOD1G93A	Amyotrophic Lateral Sclerosis	Nrf2 decreases toxicity in motor neurons	(47)
		Male Sprague–Dawley (SD) rats	Cerebral ischemia	Antioxidant effects	(45)
		Animal models, psychiatric patients	Schizophrenia, Bipolar Disorder, Depression	Restores redox balance, compensates for glutamate cycle dysfunction in astrocytes	(48)
		1RB3AN27 (N27) dopaminergic neuronal cell line	Parkinson’s	Slightly improved motor scores, oxidative stress, and mitochondrial dysfunction	(43)
		Human patients	Depression, Bipolar Disorder	Restore redox imbalance	(46)
Sulforaphane	Arugula, Asparagus, Broccoli, Cabbage	Male C57BL/6 mice	Parkinson’s	Neuronal protective effects via activating Nrf2 and mTOR	(73)
		C57BL/6 mice	Alzheimer’s	Reduced inflammation, oxidative stress; modulated Nrf2/ARE pathway	(74)
		C57BL/6 mice	Multiple sclerosis	Anti-inflammatory and anti-oxidative effects	(75)
		Male BTBR T + Itpr3tf/J (BTBR) and C57BL/6 (C57) mice	Autism Spectrum Disorder	Anti-inflammatory and antioxidant effects	(76)
		Male Sprague–Dawley rats	Vascular dementia	Suppresses neuronal loss, increases cerebral blood flow	(77)
		Chronic mild stress animal model	Depression	restores HPA axis dysfunction and anti-inflammatory	(80)
		Schizophrenia patients	Schizophrenia	Enhanced cognitive function (One Card Learning Task)	(81)
		3 × Tg-AD mouse model	Psychiatric disorder	SFN enhances the acetylation of the H3 and H4 regions of the BDNF promoter	(103)
Taurine	Shellfish (Scallops, Mussels, Clams)	Adult APP/PS1 Mouse Model	Alzheimer’s	Improvement in cognitive impairment	(113)
		Paraquat and Maneb induced PD model	Parkinson’s	Reduced inflammatory response	(108)
		Sprague–Dawley rats	Cerebral ischemia	Improves neurological function	(109)
		Male C57BL/6 J mice (Chronic Social Defeat Stress)	Depression	Reduced social avoidance, moderately improved abnormal behaviors	(111)
		Wistar rats, streptozotocin-induced diabetic model	Depression, Diabetes	antidepressant	(110)
		South Korean women (41 patients)	Major Depressive Disorder (MDD)	Concentration in hippocampus linked to MDD modulates neurotransmitter production	(112)
Sulfated Polysaccharide	Brown, Red, and Green Algae, Fucoidan	App/Ps1 transgenic mice	Alzheimer’s	Enhanced memory and cognitive abilities	(141)
		Male C57/BL6 mice	Parkinson’s	Improved mitochondrial respiratory activity, eased motor deficits	(142)
		Adult male C57BL/6 J mice (6–8 weeks)—LPS and CRS models	Depression	Antidepressant, prevent downregulation of BDNF-dependent synaptic plasticity. Improved behavioral deficits.	(143)
		Adult male Sprague–Dawley rats (260–280 g)	Depression, Stress	Antidepressant properties, reduced BDNF mRNA expression in the hippocampus	(144)
Allyl Sulfides	Garlic, Onion, Scallion, Chive, Shallot, Leek	Male BALB/c mice (FST)	Depression	SAC exhibits antidepressant-like effects, reduces hippocampus oxidative damage	(160)
		Wistar rats	Depression	Reduced malondialdehyde levels, increased SOD and GPx activity in the brain, indicating anti-depressive effects	(163)
		CSDS mice model (Male C57BL/6 J)	Depression	reduce neuroinflammation, balance oxidative stress, and decrease neuronal death in the hippocampus through NLRP3 inflammasome inhibition	(164)
Diallyl disulfide		APP-Tg mouse model	Alzheimer’s	Antioxidant, anti-inflammatory effects	(162)
Diallyl trisulfide		Transgenic human SOD1-G93A mice	Amyotrophic Lateral Sclerosis	Reduced astrocytic activation; potential toxic role in ALS	(161)
Cysteine and methionine	Egg yolks, Red bell pepper, Wheat germ, Yeast, Beans, Nuts, Cheese	5xFAD transgenic mouse model	Alzheimer’s	Antioxidant and anti-inflammatory effects	(191)
		Male Sprague–Dawley rats	Stroke model	Decreased infarct volume	(180)
		Male adult CF1 or BALB/c mice	Anxiety	reduce anxiety levels in mice during social interaction tests	(182)
		Bipolar disorder patients (Clinical Trial)	Bipolar Disorder	Reduced depressive symptoms	(183)
		Schizophrenia patients (Clinical Trial)	Schizophrenia	Reduced positive symptoms and enhanced functioning	(184)
		Cell line study	Psychiatric Disorders	potential in reducing neurotransmitter dysfunction, improving neuronal viability	(181)
		Healthy youth (age 4 to 12 years)	Autism Spectrum Disorder (ASD)	no notable effect on social impairment in adolescents with ASD	(179)
		Schizophrenia patients (*n* = 65)	Schizophrenia	improved cognition	(185)
		Schizophrenia patients (*n* = 84)	Schizophrenia	Enhance psychopathological symptoms along with improved cognitive functions	(186)
		Forty-two PD patients	Parkinson’s	Increased mitochondrial activity, reduced oxidative damage	(181)
		D-galactose-induced aging model	Aging	improved cognitive function, reduced oxidative stress and inflammation, and upregulated the transsulfuration pathway	(187)
		60 AD patients	Alzheimer’s	improvement in cognitive functions	(188)
		80 depressed postmenopausal women	Depression	improved depressive symptoms	(189)
		15 depressed patients	Major depressive disorder	effective antidepressant, improvement and recovery in depression	(190, 209)

GSH functions as a neurotransmitter and neuromodulator as it contains binding sites for putative receptors, most likely glutamate receptors (52). Although pure GSH consumption does not raise GSH levels, Jia et al. have shown that feeding curcumin (found in turmeric) to Wistar rats can boost GSH levels in the brain, and adding alpha lipoic acid (found in different vegetables) to SH-SY5Y cells can enhance intracellular GSH (53, 54). Similarly, Bruno et al. conducted an *in vitro* study on a human neuroblastoma SK-N-BE cell line and concluded that pterostilbene, a natural stilbene found in blueberries, may modulate the cell division cycle by producing advantageous alterations in DNA methylation and holds potential as a therapeutic agent for prolonging the start or further development of ND (55). Shih et al. showed the effective role of GSH produced and released by active astrocytes in maintaining the balance of synaptic redox in neurons (56). Vargas et al. demonstrated that enhanced GSH production in spinal cord astrocytes in ALS model rats suppressed the apoptosis of motor neurons (47). Another study by Kimura et al., on HT22 cells derived from the mouse hippocampus, provides evidence of the neuroprotective role of H_2_S against oxidative stress by enhancing the synthesis of GSH (57). Huang et al. showed that GSH transport from astrocytes to endothelial cells is greatly enhanced under injury conditions, and inhibiting this transport notably diminishes the protective influence of astrocytes while causing barrier dysfunction. Treatment with exogenous GSH also maintained barrier integrity without astrocytes, indicating that increasing GSH levels during disease may enhance BBB functionality (58). In addition, Song et al. demonstrated the beneficial effect of protecting motor neurons from injury by stimulating the production of GSH, decreasing the volume of brain infarcts in Sprague–Dawley rat brain following middle artery carotid occlusion. GSH may aid in improving ischemic stroke pathogenesis by reducing cerebral infarction and preventing cell death (59). Patients with depression and bipolar disorder both showed decreased levels of GSH and GPx (46). Oxidative stress and diminished antioxidant levels are prevalent in various psychiatric disorders, including schizophrenia, depression, and bipolar disorder. Enhancing glutathione synthesis aids in restoring redox imbalance in these circumstances (48). Similarly, a cross-sectional double-blind magnetic resonance spectroscopy (MRS) study using 7-Tesla magnetic resonance spectroscopy (MRS) was conducted with 57 psychosis patients and 30 healthy controls. A positive correlation was established for glutathione in the anterior cingulate cortex and social/occupational functioning. This points out that glutathione may be useful in early psychosis as a prognosis indicator (60). In a similar way, another study was conducted with 45 healthy individuals and 28 patients with schizophrenia. Using 7 T proton MRS, glutathione level was determined which was found to be lower in patients with schizophrenia than in normal healthy individuals. This observation supports the postulation of the fact that oxidative stress may well be one of the driving forces behind the disease progression (61).

### 5.2 Mechanism and molecular target of glutathione

**Figure 2:**
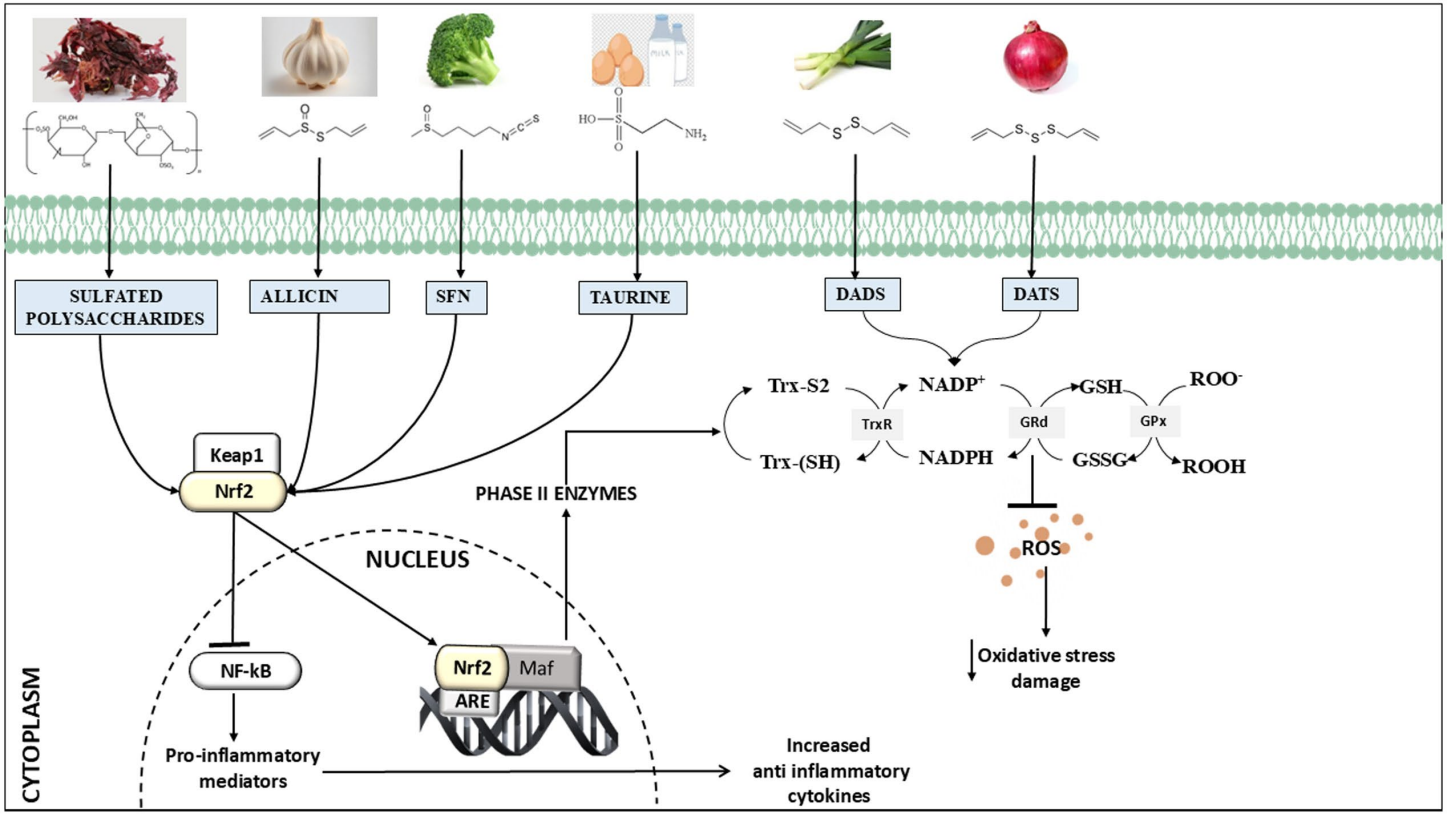
Overview of chemical structure and primary processes underlying the neuroprotective effects of natural sulfur compounds, i.e., glutathione, sulforaphane, taurine, allyl sulfides (allicin, diallyl disulfide (DADS), diallyl trisulfide (DATS), and sulfated polysaccharides), and they are recognized for their ability to activate Nrf2 (nuclear factor erythroid 2-related factor 2) and induce antioxidant benefits by upregulating ARE (antioxidant response element)-driven genes. Additionally, they have been found to reduce the inflammatory response by modulating the NFκB (nuclear factor kappa B) pathway.

GSH contains a free thiol group, which plays an important role in basal neurological regulation and cellular redox state. GSH maintains the balance of redox reactions within the cells by removing reactive nitrogen and oxygen species and also restores GSH reductase, which catalyzes the conversion of GSSG back into two GSH molecules (Figure 2). In normal settings, the intracellular ratio of reduced GSH to GSSG is more than or equal to 100. However, during oxidative stress, this ratio decreases to ≤10 (62). Excitatory amino acid carrier 1 (EAAC1) is a key protein responsible for the absorption of cysteine into neurons in rats, while in humans, this role is fulfilled by EAAT3. Furthermore, EAAC1-deficient mice exhibit age-related cognitive decline, increased oxidative stress in the hippocampus, and decreased levels of brain GSH. EAAC1-deficient rats exhibited age-related deterioration of dopaminergic neurons and elevated levels of oxidative stress (63). Another study shows that urate administration elevated its levels in the SOD1G85R-expressing Drosophila model of familial amyotrophic lateral sclerosis (fALS) by activating the Akt signaling pathway and the catalytic subunit of glutamate-cysteine ligase (64). Mitochondria are the primary source of ROS (reactive oxygen species) and RNS (reactive nitrogen species) production and contain approximately 10 to 20% of the total GSH in brain cells and most other organs (65). Because it has a similar structure to L-glutamate, which is a natural receptor agonist, and it can change its shape, it can bind to many types of glutamate receptors through its glutamyl residue (51). At low doses, GSH has a neuroprotective effect. However, at high concentrations (millimolar; mM), due to the presence of a free thiol group, the redox state of glutamate receptors can be altered (66). In context with MD, chronic social defeat stress (CSDS) is linked to a compromised glutamine-glutamate cycle in astrocytes, and studies indicate that GSH functions as a glutamate precursor when the transfer of glutamine from astrocytes to neurons is compromised. GSH depletion under stress may result from either the neutralization of ROS or an elevated requirement for glutamate precursors (67). In addition, GSH plays a role in regulating the concentration of glutamate because it can convert excessive amounts of glutamate to GSH, which would prevent excitotoxicity, a mechanism involved in diseases such as schizophrenia and ASD (46, 68).

## 6 Sulforaphane

SFN is an aliphatic isothiocyanate derived from sulfur-containing glucosinolates, which are widely recognized secondary metabolites found in plants (69). Isothiocyanates contain an isocyanate group where a sulfur atom replaces the oxygen atom, resulting in a functional group of –N=C=S (70). SFN is produced when the enzyme myrosinase acts on glucoraphanin, a glucosinolate precursor that is abundant in cruciferous vegetables such as broccoli, brussels sprouts, and cabbage (71). Sulfur-containing glucosinolates are synthesized from glucose and amino acids, which consists of the *β*-D-thioglucoside group and an N-hydroxyiminosulfate ester, leading to the formation of SFN, whereas myrosinase, an enzyme present in plants, catalyzes a hydrolysis reaction that results in the formation of SFN by joining it with the protein epithiospecifier (ESP) (inactive form). On the other hand, the active form of ESP, together with myrosinase, is responsible for the formation of SFN nitrile (72).

### 6.1 Applications of sulforaphane in animal models of mental and neurological disorders

SFN offers neuroprotection in several neurodegenerative disorders through its anti-inflammatory and antioxidant functions, as reported in Table 2 (73–82) (Table 2). SFN treatment effectively reduced memory and comprehension impairments in the 3 × Tg-AD (triple transgenic model of AD) mouse model (83). The cytoprotective role of SFN is demonstrated by a study using Aβ_25–35_ (25 μM) cytotoxicity and its interaction with Nrf2 in SH-SY5Y cell lines (84). A study was conducted in the AD model of Sprague–Dawley male rats to determine cognitive parameters post-SFN treatment, and it was found that depressive behavior and spatial learning were improved after 7 days of intraperitoneal treatment with 5 mg/kg of SFN. SFN attenuated depressive behavior through modulation of the serotonin transporter within the serotonergic system. Following a similar approach, it was demonstrated that a decreased level of SFN results in neuroinflammation and oxidative stress, as indicated by lower concentrations of malondialdehyde, TNF-*α*, and IL-1β, respectively (85). Additionally, Zhang et al. demonstrated that SFN (25 mg/kg orally) administered to C57BL/6 mice of the AD model had an improvement in both cognitive and locomotor impairments (82). Another study showed that a 90-day gavage of SFN with a combination of aluminum and D-galactose (25 mg/kg) alleviated cognitive impairments and reduced cholinergic neuron loss in MS (86). Furthermore, the authors show that SFN administration can enhance the capacity of microglial cells to engulf and remove Aβ aggregates by reducing oxidative stress (84). Various experimental studies have illustrated that SFN can modify oxidative stress and neuroinflammation in animal models of MS (87). SFN is also shown to reduce proteolytic stress by decreasing MMP-9 expression and preserving BBB, and it also elevates the concentration of anti-inflammatory cytokine IL-10 in experimental autoimmune encephalomyelitis in C57BL/6 mice (88). Dash et al. demonstrated that SFN has a notable positive effect on spatial learning and working memory post-traumatic brain injury by reducing oxidative stress (89). In addition, a study conducted in BV2 murine microglia cells, which served as a representative cell specimen for brain microglia, showed that the upregulation of antioxidant/detoxification proteins by SFN was associated with enhanced resistance of microglial cells to heat-induced toxicity and removal of ROS during microglial cell activation (90). One interesting finding indicates that SFN administration successfully reduced endothelial cell death and eliminated TJ proteins, facilitating its role in preserving the BBB (91). Another study was conducted in a streptozotocin-induced vascular dementia model in which SFN attenuates the endothelial and behavioral impairments (77). Clinical laboratory trials have shown that SFN is very well tolerated, and no serious adverse effect was observed except in rare cases of insomnia, irritability, and impaired tolerance to taste and smell (92).

Regarding MD, Yao et al. demonstrated the anti-inflammatory and antidepressant effects of SFN in a lipopolysaccharide model of depression, where a dietary intake of 0.1% glucoraphanin during juvenile and adolescent stages inhibited the development of depression-like phenotypes and alterations in synaptogenesis in adult brain regions (78). Another study indicated that C57BL/6 mice with neuropathic pain, treated intraperitoneally with repeated doses of 10 mg/kg SFN, exhibited reduced anxiety- and depressive-like behaviors associated with chronic neuropathic pain (79). A clinical trial was undertaken involving seven individuals with schizophrenia who received an oral dosage of SFN at 30 mg per day for a duration of 8 weeks. Results demonstrate that administration of SFN-rich broccoli sprout extract may improve cognitive deficits in persons with schizophrenia (81). A randomized, double-blind clinical trial was conducted with two groups: a placebo group involving 30 patients and an SFN-treated group with an equal number of participants. Compared to placebo, persons treated with SFN have exhibited superior improvements in Hamilton Rating Scale for Depression scores and increased treatment response rates (80). Furthermore, in a recent symptom-specific, placebo-controlled, double-blind, randomized trial of 172 patients with first-episode schizophrenia, the effect of sulforaphane on cognitive impairments was examined. Even though they did not demonstrate an improvement in the MATRICS Composite score, other tests concluded that spatial working memory and verbal learning have improved (*p* = 0.004 and *p* = 0.031). Indeed, the obtained results indicate that sulforaphane may have a positive impact on specific aspects of cognition in schizophrenia (93).

### 6.2 Mechanism and molecular target of sulforaphane

The mechanism and molecular target of SFN have been extensively studied in various MDs and NDs. It has been observed that SFN undergoes rapid metabolism in mammals by a process mediated by glutathione S-transferase. This mechanism creates a gradient of concentration that enables the ongoing consumption of SFN while maintaining a balance in its export (94). The Nrf2 pathway of SFN is essential for providing the neuroprotective advantages against several brain illnesses through multiple pathways and by activating results in enhanced expression of multiple downstream molecules, i.e., NAD(P)H quinone oxidoreductase-1 (NQO1), Heme oxygenase-1 (HO-1), Glutathione peroxidase-1 (GPx-1), and Gamma-glutamylcysteine synthetase (*γ*-GCS), that provide defense against oxidative stress (95). SFN functions by inhibiting and enhancing phase I enzymes interacting with cytochrome P450 and phase II enzymes by activating Nrf2 (Nuclear factor E2-factor related factor) (96) (Figure 2). Phase I enzymes perform several types of reactions, namely, oxidation, reduction, or hydrolysis, that lead to detoxification. On the other hand, phase II enzymes provide antioxidative effects and detoxifying actions by providing defense against central nervous system diseases (96). Nrf2 also regulates the expression of proteins participating in phase II detoxification processes carried out by glutathione-S-transferase and involves the association of glutathione (GSH) with xenobiotics and/or toxic materials (97). GSH is also essential for the glyoxalase system, which contributes to the detoxification of reactive dialdehydes and is utilized during protein glutathionylation, which is a crucial step in the post-translational regulation of protein metabolism (97). However, during stress, the mechanism of Nrf2 ubiquitination is hindered, and SFN can mitigate oxidative stress by stimulating the Keap1/Nrf2/ARE pathway, namely by enhancing Nrf2 activation (98). As a result, molecules including GPx1, NQO-1, HO-1, and *γ*-GCS are excessively produced, and they control the production of GSH (97, 99). Asimakopoulou et al. evaluated the role of internally produced H_2_S in the vasodilator responses triggered by SFN by using CSB/CSE inhibitors (100). These inhibitors, when administered either topically or orally, not only inhibited the cerebral vasodilatory responses but also inhibited or significantly decreased H_2_S elevations in the brain caused by both topical and oral SFN (101). Moreover, SFN is considered to possess negligible toxic effects and effectively traverses the blood–brain barrier in mice after intraperitoneal administration, potentially increasing brain-derived neurotrophic factor (BDNF) levels and enhancing dendritic spine density due to its direct impact on neurons, while also providing protection against oxidative stress via the Keap1-Nrf2 pathway (102). BDNF, a key modulator of mood and psychiatry disorders, is increased by SFN treatment in the 3 × Tg-AD mouse model. Kim et al. showed an interesting epigenetic insight into the SFN mechanism of action by enhancing the acetylation of the H3 and H4 regions of the BDNF promoter (103). Similarly, Zhao et al. experimentally showed that SFN diminishes DNA methylation at the Nrf2 promoter by enhancing Nrf2 expression (104). In addition, SFN has been found to inhibit the expression of pro-inflammatory cytokines and pathways such the TNF-*α* (tumor necrosis factor-alpha) and NF-κB (nuclear factor kappa B). These protective effects reduce inflammation of neurons in the brains of patients with conditions such as AD or PD (17, 74).

## 7 Taurine

Taurine is chemically recognized as 2-aminoethanesulfonic acid and is predominantly found in nerve and muscle tissues (105). It is a final product that can be synthesized from both methionine and cysteine, which are intermediate compounds in the transsulfuration process (106). It is produced endogenously by the enzymatic activity of cysteine dioxygenase, cysteine sulfinate decarboxylase, and hypotaurine dehydrogenase. Cysteine dioxygenase catalyzes the oxidation of cysteine, which results in cysteine sulfonic acid and subsequently hypotaurine. Hypotaurine dehydrogenase catalyzes the further oxidation of the hypotaurine produced, resulting in the formation of taurine (106, 107).

### 7.1 Applications of taurine in animal models of mental and neurological disorders

Neuroprotective effects of taurine are beneficial in several psychiatric conditions and neurodegenerative models (108–113) (Table 2). Research suggests that taurine treatments significantly improve the restoration of normal function after ischemic stroke or traumatic brain injury (114). Taurine can protect rat hippocampus and cortical neurons against the detrimental effects of Aβ in a controlled laboratory environment. Supplementation with taurine can rescue neuronal cells from glutamate-mediated excitotoxicity induced by an increased level of glutamate (109). Santa-Maria et al. showed that the majority of senile plaques in AD are composed of β-amyloid, and taurine could prevent its aggregation (115). Taurine can reduce the deterioration of dopaminergic neurons associated with neurodegenerative processes in PD, and it possesses anti-inflammatory properties that specifically target microglia. Moreover, it was shown to protect dopaminergic neurons in PD models in mice and rats by preventing microgliosis and neuroinflammation (116). Che et al. showed that the motor functions of mice were improved when taurine was administered because it reduced dopaminergic neurodegeneration and *α*-synuclein oligomerization (117). Menzie et al. have shown that by shielding brain tissue from inflammatory reactions, taurine reduced brain damage during cerebral ischemia (118). Taurine can decrease brain swelling and enhance brain function while simultaneously inhibiting nerve activity and maintaining control. It also plays a role in the growth of neurites, synapse formation, and signal transmission between neurons in the early stages of brain development (119). Furthermore, it has been demonstrated that neurons possess a fully functional taurine production pathway capable of responding to hypertonic conditions, suggesting its role in osmoregulation. As a person ages, the level of taurine in the bloodstream declines. Studies have shown that giving mice high doses of taurine (1 g/kg per day) can increase their lives by approximately 10% and enhance their overall wellbeing. This suggests that taurine may have a role in the process of aging (120). Moreover, taurine is a widely present compound, and its high concentration in the developing brain strongly indicates its vital role in neurological development (121). Wang et al. showed that taurine treatment on Sprague–Dawley rats can lead to a notable increase in cerebral blood flow of damaged ipsilateral and contralateral brain cortex at 30 min after traumatic brain injury (114). Controlled clinical trials with 24 healthy controls between 55 and 70 years of age were conducted where individuals were allocated to a control group (*n* = 11), a placebo receiving 1.5 g of starch, and a taurine group (*n* = 13), receiving 1.5 g of taurine for 16 weeks. This has shown the protective effect of taurine, indicating that it may be a potential approach for managing oxidative damage during aging as it averted the reduction of the antioxidant enzyme superoxide dismutase (122). Taurine’s role is also evident in multiple mental disease models and clinical studies: supplementation improves behavior deficits and significantly reduces social avoidance in CSDS-induced depression in C57BL/6 J mice (123). Pre-supplementation of taurine also demonstrated anti-depressant-like effects and prevented the dysregulation of neurotransmitters in a chronic unpredictable mild stress (CUMS)-induced depressive rat model (124). A streptozotocin-induced diabetic rat has low taurine in the plasma, cerebrospinal fluid, and brain, which is accompanied by depression-like behavior that was rescued by taurine supplementation (110). Clinical studies of taurine have reaffirmed its anti-depressant and anxiolytic potential. A study was conducted in South Korea with 41 young women with mild depressive disorder and 43 healthy controls. It is found that the concentration of taurine in the hippocampus may serve as a potential signal for the growth and beginning of major depressive disorder (MDD) (112). In phase II, a double-blind clinical study was conducted between 121 patients with first-episode psychosis, aged between 18 and 25 years. They were randomly assigned to consume either a placebo or 4 grams of taurine once daily for a period of 12 weeks and demonstrated improvement in the Calgary Depression Scale for Schizophrenia. The final evaluation involved 86 patients, where 47 patients received taurine while 37 received a placebo. When compared with a placebo, taurine reduces symptoms, as shown by the psychotic subscale and the BPRS (Brief Psychiatric Rating Scale) overall score. Patients with first-episode psychosis seem to benefit from adjunctive taurine in terms of psychopathology (125). Furthermore, Ohsawa et al., conducted a multicenter, phase III research that includes 10 patients with recurring stroke-like events. The dosage of taurine was based on the body weight categories of the subjects: 12 g for patients weighing 40 kg or more and 9 g for patients between 25 and 39 kg. The research indicates that taurine intake can significantly reduce the incidence of stroke-like events, and five individuals exhibited a notable enhancement by alteration of mitochondrial tRNLeu (UUR) derived from peripheral blood leukocytes (126).

### 7.2 The mechanism and molecular target of taurine

The main mechanism for the absorption of taurine in tissues is through the chloride sodium taurine transporter, and it has been found that Gamma-aminobutyric acid (GABA) transporter 2 at BBB is capable of transporting hypotaurine and taurine (127). Taurine provides potential therapeutic benefits in treating AD stem as it gives a neuroprotective role in A*β* excitotoxicity and regulates GABA receptor signaling. It improves the activity of acetylcholinesterase and acetylcholine transferase enzymes and decreases neuroinflammation induced by microglia, therefore safeguarding dopaminergic neurons (128). Hypotaurine can cross the BBB, which sets it apart from most hydrophilic molecules including GABA. The evidence that hypotaurine, through its binding to soluble Aβ, reduces the production and aggregation of oligomers, as well as its ability to lessen the toxicity of β-amyloid (Aβ) in primary cultured neurons and amyloid plaques in a mouse model, provides support for this assertion (129).

Taurine plays an important role in protecting neurons from NMDA (N-methyl-D aspartate)-induced injury by simultaneously suppressing the generation of superoxide anions. Due to its anticonvulsant properties, it reduces glutaminergic system activity while simultaneously enhancing GABAergic system activity (109). Taurine triggers the secretion of proinflammatory cytokines and stimulates the polarization of microglia towards the M1 state. In the context of AD, the inflammatory responses of M1 microglia, which are predominantly localized around the amyloid plaques, are initiated and sustained by P47 phagocyte oxidase and NF-κB (nuclear factor kappa B) (130). In the hippocampus, taurine acts as an inhibitory neurotransmitter, supplementing GABA, whose levels are known to decrease in individuals with severe MDD. Taurine plays a role in regulating the production of other neurotransmitters, such as GABA, which in turn influences mood regulation. It also possesses antioxidant and neurogenic properties (110, 131). Furthermore, taurine increases the protein level of TREM2, a receptor for Aβ and tau proteins phagocytosis by microglia. Higher levels of TREM2 are associated with reduced deposition of these neurotoxic proteins (132). Taurine functions as a neuromodulator by altering GABAergic activity. This action can be expressed in its weak agonist activity with GABA receptors; this means it helps to prevent neuronal hyperexcitability and has an anxiolytic and antidepressant effect (126, 133).

## 8 Sulfated polysaccharides

Sulfated polysaccharides (SP) are found in the cellular structure of marine algae, commonly referred to as seaweeds, in high concentrations. They are mostly composed of cellulose and hemicellulose, with a high carbohydrate content and low levels of calories and fat. Marine macroalgae, commonly referred to as seaweed, contain high amounts of sulfated polysaccharides. The majority of the seaweed cell wall consists of over 40% of SP, which is substantially higher than the average found in other sources (134). Red, brown, and green algae contain different classes of SP. Red algae include galactans, which consist of galactose units, agarans, and carrageenans, which are the types of galactans that have 4-linked *α*-galactose residues of L-series and D-series, respectively (135). The cell wall is composed of microfibrils, which consist of cellulose, ß-1, and 3-xylans. The polysaccharide found in *Porphyridium* sp. of red algae contains smaller amounts of hexuronic acid, glucuronic acid fractions, and galacturonic acid fractions, as well as galactose, xylose, glucose, and sulfate esters (136).

Marine brown algae enriched with SP are known as fucoidan which is principally made up of fucose that has a molecular weight between 20 and 200 kDa. Fucoidan mostly consists of α-L-fucose units, also known as α-L-fucopyranose (137). *Fucus vesiculosus* yielded the most basic chemical form of fucoidan, which predominantly consists of fucose, ash, and sulfate (138). Ulvan is the water-soluble polysaccharide found in green seaweed of the Ulva and *Enteromorpha* species and has a molecular weight between 150 and 2000 kDa (139). Two distinct forms of aldobiouronic acid were identified as the primary repeating disaccharide in ulvan samples. The first was ulvanobiuronic acid 3-sulfate type A, while the second was ulvanobiuronic acid 3-sulfate type B. In type A3s, the disaccharide is made up of glucuronic acid and sulfated rhamnose, but in type B3s, the major linkages are (1 → 4) glycosidic bonds between iduronic acid and sulfated rhamnose. It consists mostly of sulfate, rhamnose, xylose, glucose, iduronic acid, and glucuronic acid (140).

### 8.1 Applications of sulfated polysaccharides in animal models of mental and neurological disorders

Multiple experimental models have demonstrated the therapeutic properties of fucoidans and laminarans (141–144) (Table 2). Marine algae-derived sulfated polysaccharides have been discovered to exhibit antioxidant and anti-inflammatory characteristics (145). A study by Zhou et al. proposed that polysaccharides sourced from *Lycium barbarum* could enhance cognitive functions in APP/PS1 transgenic mice while decreasing Aβ levels (141). *Gelidium pristoides* (red alga) were exposed to Aβ1–42, resulting in the dissolution of Aβ1–42 fibrils, suggesting that the polysaccharides possess the capacity to disassemble and hinder the creation of fibrils. The findings indicate that these properties of sulfated polysaccharides could be further investigated for their use as nutraceuticals in the treatment of ND (146). Fucoidan enhanced the functioning of mitochondria and protected the brain by actively controlling the Nrf2 pathway. Administering fucoidan isolated from *L. japonica* at a dosage of 2 mL/kg/d to mice with PD induced by rotenone showed a protective impact on mitochondrial activity and the degeneration of dopaminergic neurons (147). Xing et al. investigated the neuroprotective effect of type II fucoidan from *Fucus vesiculosus* in male C57BL/6 mice by not only safeguarding against neurodegeneration but also preserving substantia nigra activity and alleviating mitochondrial dysfunction. Moreover, it reduces movement impairments in the 1-methyl-4-phenyl-1,2,3,6-tetrahydropyridine-induced PD mouse model by acting on the ATP5F1a protein (142). In a recent study, Nambi et al. demonstrated that administration of Fucoidan along with cerebrolysin in adult male Sprague–Dawley rats in cerebral ischemia model results in substantial decrease in neurological impairments and cerebral infarct volume (148).

Furthermore, in psychiatric disease models, Li et al. showed that fucoidan could help adult male (6–8 weeks) C57BL/6 J mice who were depressed: those who were exposed to lipopolysaccharide (LPS) and those who were under chronic restraint stress. Acute administration of fucoidan did not yield an antidepressant effect, whereas dose-dependent chronic fucoidan supplementation mitigated stress-induced depressive-like behaviors. Interestingly, chronic supplementation with fucoidan mitigated the downregulation of BDNF-dependent synaptic plasticity by reducing caspase-1-mediated inflammation and substantially improving behavioral deficits associated with caspase-1 overexpression in the hippocampus of mice. Moreover, the inhibition of BDNF eliminated the depressive-like behavioral effects of fucoidan in mice (143). Researchers conducted a study on 260–280-g adult male Sprague–Dawley rats. They gave the rats fucoidan intraperitoneally at different doses for 30 min before exposing them to repeated restraint stress twice a day for 14 days. Fucoidan significantly inhibited depressive-like behavior and reduced BDNF mRNA expression in the hippocampus. Overall findings indicate that administering fucoidan prior to restraint stress significantly decreased helplessness behavior in rats, possibly through changes in the central noradrenergic system (144). A randomized, placebo-controlled clinical trial was conducted on 86 subjects aged 18 to 65 years, divided into two groups, one receiving ulva extract and the other receiving a placebo. Findings showed a significant reduction in depressive symptoms compared to the placebo group and improvements in sleep disorders and psychomotor functions. This study indicates that *Ulva lactuca* may provide a natural option for the management of depression, potentially evading the negative effects linked to traditional antidepressants (149). A separate clinical trial investigated the impact of marine algae extracts on depression-associated behaviors in animal models. The results showed that these extracts greatly improved symptoms of depression, supporting the idea that metabolites found in marine algae such as ulvans may have antidepressant properties (150).

### 8.2 The mechanism and molecular target of sulfated polysaccharides

By inhibiting inflammatory pathways and reducing the release of pro-inflammatory molecules, natural sulfur compounds may help alleviate neuroinflammation linked to several NDs. Sulfated polysaccharides, including fucoidans from *Sargassum fusiform*, loliolide from *Codium tomentosum* (green seaweeds), and phycoerythrin from *Gracilaria gracilis* (red seaweeds), exhibit antioxidant properties by efficiently counteracting detrimental free radicals and preventing lipid oxidation (151). Phenolic compounds work as antioxidants and protect neurons from oxidative damage, preserving their structural and functional integrity (152). Brown algae possess a notable affinity for heavy metals, enabling them to form stable metal complexes via transition-metal chelation. Phenolic compounds possess strong chelating properties that directly impede the generation of reactive OH free radicals from the Fenton process by attaching to Fe3+ metal ions (153). These metal chelators possess the capability to cross the BBB and so may be appropriate for treating NDs. Fucoidan protects the nerve cells against oxidative stress damage through the suppression of cytochrome c release from the mitochondria to the cytosol and modulates anti-apoptosis gene activation. Moreover, fucoidan also increases BDNF and modulates synaptic plasticity (154). Furthermore, sulfated polysaccharides have shown an ability to inhibit cholinesterase activity, which is useful in diseases such as AD. Similar to anti-acetyl cholinesterase compounds, sulfated polysaccharides prolong the duration of action of acetylcholine, a neurotransmitter important for memory and cognition. It assists in enhancing the signal crossing strength and information processing abilities in AD models (155).

## 9 Allyl sulfides

Allyl sulfide is an organosulfur compound with two functional groups: an allyl and a sulfide. They are a significant class of organic compounds commonly found in members of the Allium family, such as garlic and onions. These compounds are responsible for their pungent smell and health benefits. Allyl sulfides are compounds that contain an allyl group (a three-carbon chain with a double bond, CH₂ = CH-CH₂−) bonded to one or more sulfur atoms. The simplest form is diallyl sulfide (DAS), which contains one sulfur atom linked to two allyl groups and is less reactive than the others. Diallyl disulfide (DADS) consists of a disulfide bond that is more reactive than DAS, whereas diallyl trisulfide (DATS), a complex structure of three sulfur atoms, is more reactive due to the presence of multiple sulfur atoms, which increases its capacity to engage in redox reactions (156). Allyl sulfides are lipophilic thioesters formed from allicin found in garlic and other Allium vegetables (157). Garlic, when consumed, activates the allinase enzyme, which speeds up the conversion of alliin to allicin, an unstable metabolite that swiftly disintegrates into four H_2_S-releasing compounds due to its instability in aqueous media (158). Among them, DATS is the most active inducer of phase II enzyme gene expression (152). These organosulfur compounds are responsible for biological functions such as antioxidant and anti-inflammatory, which can be attributed to their chemical structure and function as neuroprotective agents (159).

### 9.1 Applications of allyl sulfides in animal models of mental and neurological disorders

Evidence suggests that consuming Allium vegetables such as garlic and onions as a functional food and traditional herbs can lead to enhanced antioxidant and anti-inflammatory activity (160–164) (Table 2). A recent study found that administering DADS at doses of either 40 or 80 mg/kg produced therapeutic effects comparable to those of imipramine, a prescription antidepressant at a dose of 10 mg/kg, in treating mice with depression-like behaviors induced by LPS (160). Moreover, DADS enhances synaptic plasticity, and this improvement could potentially enhance learning and memory in AD (165). Guo et al. revealed the importance of heme oxygenase 1 (HO-1) where a significant rise in HO-1 synthesis was observed in SOD1-G93A mice treated with DATS compared to animals that received a placebo. These data indicate that the administration of DATS prolongs the lifespan of SOD1-G93A transgenic mice and suppresses astrocytic activation. Thus, DATS is a very probable neuroprotective agent for ALS (161). AGE (aged garlic extract), when given orally, can enhance spatial recognition memory in rats with cognitive impairment caused by Aβ42 in AD. This extract is devoid of smell and contains powerful antioxidant components that efficiently decrease oxidative harm (162). In mice subjected to ischemia–reperfusion, the injection of DATS led to a reduction in the concentration of MMP-9 and oxidative stress. This reduction contributed to a decrease in BBB leakage and vasogenic edema (166). In a recent study, it was revealed that in the ischemic stroke model in C57 mice, the intervention of allicin has the potential to decrease areas of cerebral infarction (167).

Ruiz-Sánchez found that S-allyl cysteine has an antidepressant-like effect and reduces oxidative damage to the hippocampus in the FST model (168). In addition, a study conducted in 56 male Wistar rats receiving garlic homogenate at different doses of 0.1, 0.25, and 0.5 g/kg for 10 days showed improvement in depression-related behaviors evaluated by the forced swim test and elevated plus maze (163). Another study showed that supplementing garlic at different doses improved social interaction in a CSDS mouse model (164).

### 9.2 The mechanism and molecular target of allyl sulfides

Studies indicate that diallyl sulfides can activate drug-metabolizing enzymes such as NQO1 and HO-1 in a manner that depends on the Nrf2/ARE pathway. This notion is corroborated by the animal investigations where DATS was found to stimulate the expression of various detoxification enzyme genes in normal mice but not in mice lacking the Nrf2 gene (111). A study indicated that the activation of both ERK and p38MAPK pathways is crucial in the process of Nrf2 nuclear translocation and HO-1 gene activation triggered by DAS (169). In contrast, a separate investigation discovered that DATS stimulated MAPKs while inhibiting MAPKs did not impact the ARE activity generated by DATS (111). The study found that the PKC pathway was not directly responsible for the activation of ARE by DATS. It also suggested that Nrf2 may not be the sole transcription factor or signaling molecule involved in the cytoprotective signals initiated by DATS. However, the calcium-dependent signaling pathway seemed to contribute to the cytoprotective effect induced by DATS (111).

A recent study in animals has shown that DADS operates inside the H_2_S/BDNF/Nrf2 pathway, which suppresses neuropathic pain (170). Nrf2 was found to suppress the various downstream proinflammatory cytokines IL-6 and IL-1β. In mice with normal genes, the presence of mutant tau in the hippocampus results in an upregulation of HO-1 and GCLC transcripts. However, this effect is not observed in animals lacking the Nrf2 gene. This indicates that Nrf2 is crucial in diminishing oxidative stress and inflammation (171). TAR DNA binding protein 43 (TDP-43) is a marker that has been identified as both a pathological and biochemical indicator in ALS (172). It has been demonstrated that the administration of DATS effectively inhibited the rise in ROS level caused by the expression of TDP-43 and enhanced cell viability by promoting the Nrf2 pathway. This suggests that using this molecule as a therapeutic approach in ALS could be beneficial (173). Moreover, garlic mitigates anxiety and depressive behaviors in diabetic rats, potentially through the reduction of oxidative stress in the brain (163). Allicin mitigated depression-like behaviors in CSDS mice by diminishing neuroinflammation, equilibrating oxidative stress, and lowering neuronal death in the hippocampus by inhibiting the NLRP3 inflammasome (164).

## 10 Cysteine and methionine

Cysteine is a sulfur-containing amino acid that can be either obtained from dietary sources or synthesized via the transsulfuration pathway. The thiol group of cysteine commonly acts as a nucleophile in enzymatic processes. Cystine, the most prevalent form of cysteine, exhibits plasma concentrations that are 10-fold greater than cysteine (174). Cysteine is classified as one of the amino acids that have a polar and uncharged R group. This R group is more hydrophilic compared to amino acids that have a non-polar side chain (175). Cysteine exhibits chirality, and both D-cysteine and L-cysteine occur naturally with D-cysteine being detected in the developing brain, and L-cysteine is the predominant type of cysteine found in our body (176). Cysteine is synthesized via the enzymatic activity cystathionase/cystathionine *γ*-lyase (CSE) acting on cystathionine. Cystathionine, on the other hand, is formed by the combination of homocysteine and serine through the enzyme cystathionine *β*-synthase (CBS). Recent investigations have shown the neuroprotective role of CSE. The depletion of CSE leads to oxidative stress and abnormal stress responses (177). Methionine is another sulfur-containing amino acid present in our diet. It acts as a beginning point in the methionine cycle and a precursor of S-adenosylmethionine (SAMe), a vital molecule involved in several biochemical processes. Upon metabolism into SAMe, it donates its methyl group and is converted into S-adenosylhomocysteine (SAH). SAH is then hydrolyzed to homocysteine, a key intermediate in the cycle. Homocysteine can either be remethylated back into methionine or redirected to the TSP to form cysteine, a precursor for glutathione. Methionine cycle intermediate, SAMe, demonstrates antioxidant properties, influences DNA methylation, elevates GSH levels, and, at elevated doses, mitigates neuronal loss in NDs (178).

### 10.1 Applications of cysteine and methionine in animal models of mental and neurological disorders

Studies have observed low levels of cysteine in both autism spectrum disorder (ASD) patients and control subjects following an overnight period of fasting. These findings indicate the correlation between high levels of oxidative stress and low detoxifying capacity in individuals with ASD (179–191). Monti et al. demonstrated that oral doses with intravenous administration of N-acetylcysteine (NAC) at particular doses for 3 months have a protective effect against damage to dopaminergic terminals in Parkinson’s model. Prior administration of NAC in animal models that have been subjected to intracerebroventricular administration of Aβ has resulted in an enhancement in learning and memory (192). Khan et al. noted that administering NAC after the occurrence of ischemia decreased the size of the infarction and enhanced the neurologic score. The administration of NAC resulted in a more pronounced reduction in infarct volume in the cortex and striatum, as evidenced by a previous study (180). It is shown that NAC partially enhanced the endurance of hippocampal neurons following temporary forebrain ischemia (193). During an *in vivo* investigation involving transgenic APP/PS-1 mice, the oral administration of NAC through drinking water prior to the development of the disease demonstrated the ability to reduce oxidative damage in neurons (174). Administration of NAC after the onset of ischemia in rat stroke model proved its neuroprotective effect by decreasing the infarction size and enhancing the neurologic score (180). Louapre et al. suggest that NAC may modify cerebral blood flow in specific brain regions, such as the frontal and temporal lobes, which are associated with enhancements in cognitive function among individuals with MS (194). Similarly, external supplementation with NAC restores cysteine level, which decreased during hypobaric hypoxia, eliminates endogenous hydrogen sulfide concentrations, and reduces neuronal dysfunction (195). A clinical study in randomized 23 MS patients who had received NAC intravenously on a weekly basis and then orally for the next 6 days for 2 months has revealed that whole brain CBF showed a substantial rise after NAC treatment (196). Notably, neurotransmitter dysfunction is apparent across the range of psychiatric disorders. In a cell line study, NAC has shown the potential to reduce the dysfunction of both dopamine and glutamate by enhancing dopamine receptor binding and neuronal viability (181). Another study administered 60–150 mg/kg NAC intraperitoneally to male adult CF1 or BALB/c mice every day for 4 days, 1 h before social interaction tests. This suggests that NAC treatment reduces anxiety levels (182). Furthermore, clinical studies are also encouraging a randomized, double-blind, controlled trial conducted with 75 bipolar disorder patients for 24 weeks. Patients were assigned to receive once-daily NAC 2 g for 4 weeks and demonstrated reduced depression symptoms, while the placebo group experienced more severe signs of depression at the end of the trial (183). A clinical trial involving 121 patients examined the impact of NAC on schizophrenia, treating 59 with NAC and 62 with a placebo. The results show that additive NAC may help patients more than a placebo in improving their functioning and lowering their positive symptoms. This could lead to the suggestion of stage-specific therapies (184). Another randomized, double-blind, placebo-controlled trial examined 65 schizophrenia patients who received NAC (1.8 g + 0.9 g) orally twice a day for 6 months. Giving NAC to a small group of early psychosis patients did not make their negative symptoms worse, but it did improve their cognition and raise GSH levels in the medial prefrontal cortex of their brains (185). Similarly, a placebo-controlled clinical trial was conducted with 84 schizophrenia patients who received NAC orally twice daily (0.6 g) for 12 weeks. This study observed enhancements in positive and negative psychopathological symptoms and cognitive functions (186). Physiological application of methionine supplementation is dose-dependent, for instance, a low methionine diet suppresses the neurogenesis in animal models, whereas high dietary methionine has been associated with mild cognitive impairment primarily due to mitochondrial dysfunction (197). Moreover, D-galactose-induced aging model found that methionine intake improved cognitive function, reduced oxidative stress and inflammation, and upregulated the TSP. These improvements were linked to enhance BDNF–TrkB signaling and modulation of neuronal signal transmission (187). SAMe clinical efficacy is demonstrated in multiple clinical studies such as AD and depression. For instance, SAMe, in a double-blind, placebo-controlled study of 60 AD patients who received a daily dose of 400 mg for 180 days, reported improved cognitive functions (188). Similarly, Salmaggi et al. showed that 1,600 mg SAMe received orally by depressed postmenstrual women has resulted in significantly improved depressive symptoms (189). Another single-blind study involving depressed patients indicated improvement or recovery in seven out of nine participants. The reaction to the antidepressant was fast and easily tolerated (190).

### 10.2 Mechanism and molecular target of cysteine and methionine

The metabolism of homocysteine involves two pathways, i.e., reverse transsulfuration and transmethylation pathway, which results in its conversion into cysteine and methionine, respectively (Figure 1). Due to the impact of folate obtained from diet and vitamin B12 on the incorporation of homocysteine into the reverse TSP, the concentrations of these cofactors may regulate the formation of H_2_S (198). Vitamin B6 deficiency modulates cysteine synthesis, and it has been observed that supplementation with vit B6 was found to be beneficial because the enzymes CSE and CBS rely on pyridoxal 5-phosphate as a cofactor affects the flow through the reverse TSP. The enzyme CSE catalyzes the synthesis of cysteine from cystathionine, and its promoter contains a binding site for Nrf2, and expression can be triggered by oxidative stress (199). An investigation documented the process of sulfhydration of Keap1, which subsequently triggers the activation of Nrf2, which is the main regulator of the response to oxidative stress. Nrf2 has been documented to possess binding sites known as antioxidant response elements for the upstream regions of CBS and CSE. Therefore, Nrf2 can increase the expression of both CSE and CBS (200). Oxidative stress triggers the activation of CSE, which is a crucial step in a pathway that ultimately results in the production of two important antioxidants: cysteine and GSH. These antioxidants play a key role in mediating the cellular response to oxidative stress by sulfhydrating proteins implicated in this response (201).

The significance of the X_c_^−^ cystine–glutamate exchanger (SLC7A11 carrier) in the cysteine redox cycle, which regulates the extracellular redox potential is becoming now more widely acknowledged (202). Inhibition of the X_c_^−^ exchanger inhibits the growth and promotes the death of glioma cell lines. In research conducted on the AD model of C57BL/6 mice and Wistar rats, it has been observed that activated microglia exhibit enhanced expression of the X_c_^−^ exchanger associated with higher secretion of glutamate (203). During exposure of cultured neurons to activated microglia, the toxicity of amyloid beta peptide 1–40 was enhanced by the release of glutamate through the X_c_^−^ exchanger. Therefore, by inhibiting NMDA receptors or system X_c_^−^ the toxicity of peptide mediated by microglia was prevented (203).

Investigations have shown that xCT expression is increased in both the spinal cord of ALS patients and isolated microglia of mutant superoxide dismutase 1 (SOD1) ALS animals. Furthermore, the expression of xCT is associated with upregulation of inflammation and increased release of glutamate (204). Methionine is an essential component of one-carbon metabolism, where it gets converted to SAMe. This conversion is essential to the methylation of cytosine in CpG islands that exist in the promoters of genes to modulate the expression of a gene by affecting the binding of transcription factors to DNA. There should be equal amounts of SAMe and SAH present; high SAH may inhibit DNA methyltransferases (DNMTs), thus reducing methylation and causing possible uncontrolled gene expression (205). Furthermore, the ratio of methionine to homocysteine can be seen as a new imperative biomarker of the dementia risk; the enhanced ratio leads to better cognitive performances and slow brain atrophy over the course of the disease (206). Hyperhomocysteinemia (HHcy), an elevated level of homocysteine, can influence the hypomethylation of some of the most sensitive genes, which catalyze numerous disorders and ND, such as AD. Moreover, homocysteine levels can be reduced by including supplements such as vitamin B (e.g., folate), which has been reported to reestablish normalcy in DNA methylation, hence providing an evidence-based perspective towards the treatment of diseases related to HHcy (207).

## 11 Conclusion and future perspectives

Several studies have indicated that an imbalance between oxidative stress and antioxidant defense mechanisms is linked to the onset of ND and MD. This imbalance results in the formation of free radicals and the degradation of lipids and proteins, ultimately leading to substantial death of neurons. The possible neuroprotective effects of sulfur-containing compounds found in foods such as cruciferous vegetables, garlic, onions, and some seafood have been attributed to various antioxidant mechanisms. Some of these compounds can chelate free radicals and augment transcription machinery by Nrf2 and ROS, thereby depriving them of the ability to induce oxidative stress, a condition that is associated with neuronal pathology.

Additionally, compounds that contain sulfur can influence various pathways in cells that relate to inflammation and programmed cell death which, in turn, helps preserve neurons. Interestingly, some of the natural sulfur compound such as cysteine, GSH, and taurine are also intermediates of TSP, which is a fundamental metabolic pathway that maintains the redox buffer in cells. Sulfur-containing amino acid, such as cysteine, is critical in the brain’s ability to maintain redox buffering. Another component involved in this pathway is GSH, whose precursor is cysteine, which is significant in maintaining the intracellular redox state and preventing neurons from being damaged by free radicals. Hence, GSH acts as an antioxidant by trapping ROS and ensuring the body has optimum function at the cellular level. It is plausible that dietary alterations involving sulfur-containing compounds could also increase the activity of the TSP and increase the GSH levels. Notably, another H_2_S transulfuration intermediate, taurine, can also be obtained by diet. It has also been found that H_2_S is vital in maintaining the redox reaction in the brain and stimulates strong antioxidant effects. H_2_S reduces oxidative stress by enhancing the concentration of cysteine imported into neurons by the cysteine transporter and the cystine/glutamate antiporter. This, in turn, enhances the synthesis of glutathione, which is a potent antioxidant. Redox imbalance associated with altered H_2_S has been found in many MDs and NDs. However, H_2_S is an endogenous gasotransmitter, and accurately controlling its effective concentration *in vivo* is challenging. This difficulty limits using H_2_S gas as a drug in basic research and clinical trials. This challenge has prompted the exploration of natural sulfur compounds, which can increase the sulfur pool in the cell in a more controlled and sustained way, providing a more effective solution for therapeutic applications. Therefore, natural sulfur compounds can be promising alternatives to H_2_S as therapeutic agents.

Moreover, in the context of both MD and ND, the nutritional advantages of foods high in sulfur offer a promising anti-inflammatory intervention. Foods rich in sulfur can help the anti-inflammatory mechanism and may even act as a hindrance in the pace of the development of neuropathology. Compounds that contain sulfur have been established to give anti-inflammatory effects due to their action in the antioxidant and inflammation signal transduction in the brain. It can, therefore, be argued that integrating foods rich in sulfur into dietary plans is a worthy approach to combating brain-associated diseases. More empirical studies, including clinical trials, are required to completely comprehend the possible advantages and modes of action. Individual differences in food, heredity, and the course of the disease may also affect how the body reacts to substances that contain sulfur. Therefore, in the overall management of a broad spectrum of brain diseases, including ND and MD, sulfur-based interventions should be viewed as supplemental tactics to well-established medical treatments and lifestyle changes.

However, the challenges in preclinical animal study should be carefully considered before evaluating the efficiency of natural sulfur compounds. Animal models often have low predictive validity for human diseases. Notably, in neuropsychiatry pharmacology only, 9% of preclinical findings have proven effective clinically (208). Animal models also have diverse microflora, which could be another reason for ambiguity in preclinical results. Future research should address the discrepancy between the doses administered in animal studies and the corresponding dose levels in humans. Random errors are another concern in animal studies, as only few studies fully comply with the basic principles of research design. This requires more pharmacological and pharmacokinetic data across the animal models to conclude. Nevertheless, the preclinical animal model gives proof-of-concept and is pivotal for designing clinical trials and successfully generating a wealth of data in drug discovery. Furthermore, multiple evidence suggest that alternation in epigenetics regulations, i.e., histone modification and DNA methylation, contribute significantly to pathological diseases and NDs. Natural sulfur compounds such as GSH, SFN, taurine, allyl sulfide, sulfated polysaccharides, and cysteine and methionine having roles in modulating histone deacetylases, DNA methyltransferases, and modulating SAMe expression are also emerging as potential interventions under these conditions besides its major role in anti-inflammatory and oxidative stress.

To summarize, the reviewed research indicates that foods rich in natural sulfur compounds offer a valuable source of nutrients that possess neuroprotective properties against neuropathology. Further research is necessary to comprehensively comprehend the metabolism and precise mechanisms of action to optimize dietary habits for maximizing the advantages of natural sulfur compounds in promoting brain health.

## References

[1] Oae S. (ed.). Organic Chemistry of Sulfur. Boston, MA: Springer US. (1977).

[2] Huxtable RJ. Biochemistry of Sulfur. Boston, MA: Springer US. (1986).

[3] Francioso A Baseggio Conrado A Mosca L Fontana M. Chemistry and biochemistry of sulfur natural compounds: key intermediates of metabolism and redox biology. Oxidative Med Cell Longev. (2020) 2020:8294158–27. doi: 10.1155/2020/8294158, PMID: 33062147 PMC7545470

[4] Künstler A Gullner G Ádám AL Kolozsváriné Nagy J Király L. The versatile roles of sulfur-containing biomolecules in plant defense—a road to disease resistance (2020) 9:1705. doi: 10.3390/plants9121705 PMC7761819 33287437

[5] Sies H Berndt C Jones DP. Oxidative stress. Annu Rev Biochem. (2017) 86:715–48. doi: 10.1146/annurev-biochem-061516-045037, PMID: 28441057

[6] Vitvitsky V Thomas M Ghorpade A Gendelman HE Banerjee R. A functional Transsulfuration pathway in the brain links to glutathione homeostasis. J Biol Chem. (2006) 281:35785–93. doi: 10.1074/jbc.M602799200 17005561

[7] Paul BD. Neuroprotective roles of the reverse Transsulfuration pathway in Alzheimer's disease. Front Aging Neurosci. (2021) 13:659402. doi: 10.3389/fnagi.2021.659402, PMID: 33796019 PMC8007787

[8] Panthi S Chung HJ Jung J Jeong NY. Physiological importance of hydrogen sulfide: emerging potent Neuroprotector and neuromodulator. Oxidative Med Cell Longev. (2016) 2016:9049782. doi: 10.1155/2016/9049782 PMC4931096 27413423

[9] Petropoulos S Di Gioia F Ntatsi G. Vegetable organosulfur compounds and their health promoting effects. Curr Pharm Des. (2017) 23:2850–75. doi: 10.2174/1381612823666170111100531, PMID: 28078991

[10] Irwin MI Hegsted DM. A conspectus of research on amino acid requirements of man. J Nutr. (1971) 101:539–66. doi: 10.1093/jn/101.4.539, PMID: 4931584

[11] Sbodio JI Snyder SH Paul BD. Regulators of the Transsulfuration pathway. Br J Pharmacol. (2019) 176:583–93. doi: 10.1111/bph.14446 30007014 PMC6346075

[12] Steegborn C Clausen T Sondermann P Jacob U Worbs M Marinkovic S. Kinetics and inhibition of recombinant human cystathionine gamma-Lyase. Toward the rational control of Transsulfuration. J Biol Chem. (1999) 274:12675–84. doi: 10.1074/jbc.274.18.12675, PMID: 10212249

[13] Jefferies H Coster J Khalil A Bot J McCauley RD Hall JC. Glutathione. ANZ J Surg. (2003) 73:517–22. doi: 10.1046/j.1445-1433.2003.02682.x 12864828

[14] Zhang H Forman HJ. Glutathione synthesis and its role in redox signaling. Semin Cell Dev Biol. (2012) 23:722–8. doi: 10.1016/j.semcdb.2012.03.017 22504020 PMC3422610

[15] Flagg EW Coates RJ Eley JW Jones DP Gunter EW Byers TE. Dietary glutathione intake in humans and the relationship between intake and plasma Total glutathione level. Nutr Cancer. (1994) 21:33–46. doi: 10.1080/01635589409514302 8183721

[16] Al-Temimi AA Al-Mossawi AE Al-Hilifi SA Korma SA Esatbeyoglu T Rocha JM. Glutathione for food and health applications with emphasis on extraction, identification, and quantification methods: A review. Meta. (2023) 13:465. doi: 10.3390/metabo13040465 PMC10141022 37110125

[17] Schepici G Bramanti P Mazzon E. Efficacy of Sulforaphane in neurodegenerative diseases. Int J Mol Sci. (2020) 21:8637. doi: 10.3390/ijms21228637, PMID: 33207780 PMC7698208

[18] Alfieri A Srivastava S Siow RCM Cash D Modo M Duchen MR. Sulforaphane preconditioning of the Nrf2/ho-1 defense pathway protects the cerebral vasculature against blood-brain barrier disruption and neurological deficits in stroke. Free Radic Biol Med. (2013) 65:1012–22. doi: 10.1016/j.freeradbiomed.2013.08.190 24017972

[19] Nandini DB Rao RS Deepak BS Reddy PB. Sulforaphane in broccoli: the green chemoprevention!! Role in Cancer prevention and therapy. J Oral Maxill Pathol. (2020) 24:405. doi: 10.4103/jomfp.JOMFP_126_19, PMID: 33456268 PMC7802872

[20] Ripps H Shen W. Review: taurine: A "very essential" amino acid. Mol Vis. (2012) 18:2673–86. PMID: 23170060 PMC3501277

[21] Rafiee Z García-Serrano AM Duarte JMN. Taurine supplementation as a neuroprotective strategy upon brain dysfunction in metabolic syndrome and diabetes. Nutrients. (2022) 14:1292. doi: 10.3390/nu14061292 35334949 PMC8952284

[22] Santulli G Kansakar U Varzideh F Mone P Jankauskas SS Lombardi A. Functional role of taurine in aging and cardiovascular health: An updated overview. Nutrients. (2023) 15:4236. doi: 10.3390/nu15194236, PMID: 37836520 PMC10574552

[23] Pan Z Agarwal AK Xu T Feng Q Baerson SR Duke SO. Identification of molecular pathways affected by Pterostilbene, a natural Dimethylether analog of resveratrol. BMC Med Genet. (2008) 1:7. doi: 10.1186/1755-8794-1-7 PMC2330146 18366703

[24] Hansen SH Andersen ML Cornett C Gradinaru R Grunnet N. A role for taurine in mitochondrial function. J Biomed Sci. (2010) 17:S23. doi: 10.1186/1423-0127-17-s1-s23 20804598 PMC2994382

[25] Gruhlke MC Slusarenko AJ. The biology of reactive sulfur species (Rss). Plant Physiol. Biochem. (2012) 59:98–107. doi: 10.1016/j.plaphy.2012.03.016, PMID: 22541352

[26] Tateishi N Higashi T Naruse A Hikita K Sakamoto Y. Relative contributions of sulfur atoms of dietary cysteine and methionine to rat liver glutathione and proteins. J Biochem. (1981) 90:1603–10. doi: 10.1093/oxfordjournals.jbchem.a133635 7333998

[27] Moskaug J Carlsen H Myhrstad MC Blomhoff R. Polyphenols and glutathione synthesis regulation. Am J Clin Nutr. (2005) 81:277s–83s. doi: 10.1093/ajcn/81.1.277S 15640491

[28] Minich DM Brown BI. A review of dietary (Phyto)nutrients for glutathione support. Nutrients. (2019) 11:2073. doi: 10.3390/nu11092073 31484368 PMC6770193

[29] Matusheski NV Jeffery EH. Comparison of the bioactivity of two glucoraphanin hydrolysis products found in broccoli, Sulforaphane and Sulforaphane nitrile. J Agric Food Chem. (2001) 49:5743–9. doi: 10.1021/jf010809a 11743757

[30] Farnham M Stephenson K Fahey J. Glucoraphanin level in broccoli seed is largely determined by genotype. HortScience. (2005) 40:50–3. doi: 10.21273/HORTSCI.40.1.50

[31] Asif Ali M Khan N Kaleem N Ahmad W Alharethi SH Alharbi B. Anticancer properties of Sulforaphane: current insights at the molecular level. Front Oncol. (2023) 13:1168321. doi: 10.3389/fonc.2023.1168321, PMID: 37397365 PMC10313060

[32] Demirkol O Adams C Ercal N. Biologically important thiols in various vegetables and fruits. J Agric Food Chem. (2004) 52:8151–4. doi: 10.1021/jf040266f 15612810

[33] Kasamatsu S Kinno A Hishiyama J Akaike T Ihara H. Development of methods for quantitative determination of the total and reactive polysulfides: Reactive polysulfide profiling in vegetables. Food Chem. (2023) 413:135610. doi: 10.1016/j.foodchem.2023.135610 36774840

[34] Nicastro HL Ross SA Milner JA. Garlic and onions: their Cancer prevention properties. Cancer Prev Res (Phila). (2015) 8:181–9. doi: 10.1158/1940-6207.Capr-14-0172 25586902 PMC4366009

[35] Szymański K Winiarska K. Taurine and its potential therapeutic application. Postepy higieny i medycyny doswiadczalnej. (2008) 62:75–86. 18305447

[36] Wu G. Important roles of dietary taurine, Creatine, carnosine, anserine and 4-Hydroxyproline in human nutrition and health. Amino Acids. (2020) 52:329–60. doi: 10.1007/s00726-020-02823-6 32072297 PMC7088015

[37] Wójcik OP Koenig KL Zeleniuch-Jacquotte A Costa M Chen Y. The potential protective effects of taurine on coronary heart disease. Atherosclerosis. (2010) 208:19–25. doi: 10.1016/j.atherosclerosis.2009.06.002 19592001 PMC2813349

[38] Bixler HJ Porse H. A decade of change in the seaweed hydrocolloids industry. J Appl Phycol. (2011) 23:321–35. doi: 10.1007/s10811-010-9529-3, PMID: 40082651

[39] Muthukumar J Chidambaram R Sukumaran S. Sulfated polysaccharides and its commercial applications in food industries-a review. J Food Sci Technol. (2021) 58:2453–66. doi: 10.1007/s13197-020-04837-0 34194082 PMC8196116

[40] Hentati F Tounsi L Djomdi D Pierre G Delattre C Ursu AV. Bioactive polysaccharides from seaweeds (2020) 25:3152. doi: 10.3390/molecules25143152, PMID: 32660153 PMC7397078

[41] Franco R Schoneveld OJ Pappa A Panayiotidis MI. The central role of glutathione in the pathophysiology of human diseases. Arch Physiol Biochem. (2007) 113:234–58. doi: 10.1080/13813450701661198 18158646

[42] Forman HJ Zhang H Rinna A. Glutathione: overview of its protective roles, measurement, and biosynthesis. Molecul Aspects Med. (2009) 30:1–12. doi: 10.1016/j.mam.2008.08.006 PMC2696075 18796312

[43] Harish G Venkateshappa C Mythri RB Dubey SK Mishra K Singh N. Bioconjugates of curcumin display improved protection against glutathione depletion mediated oxidative stress in a dopaminergic neuronal cell line: implications for Parkinson's disease. Bioorg Med Chem. (2010) 18:2631–8. doi: 10.1016/j.bmc.2010.02.029 20227282

[44] Dean O Bush AI Berk M Copolov DL van den Buuse M. Glutathione depletion in the brain disrupts short-term spatial memory in the Y-maze in rats and mice. Behav Brain Res. (2009) 198:258–62. doi: 10.1016/j.bbr.2008.11.017 19061918

[45] Higashi Y Aratake T Shimizu T Shimizu S Saito M. Protective role of glutathione in the Hippocampus after brain ischemia. Int J Mol Sci. (2021) 22:7765. doi: 10.3390/ijms22157765 34360532 PMC8345998

[46] Poladian N Navasardyan I Narinyan W Orujyan D Venketaraman V. Potential role of glutathione antioxidant pathways in the pathophysiology and adjunct treatment of psychiatric disorders. Clin Pract. (2023) 13:768–79. doi: 10.3390/clinpract13040070, PMID: 37489419 PMC10366746

[47] Vargas MR Pehar M Cassina P Beckman JS Barbeito L. Increased glutathione biosynthesis by Nrf2 activation in astrocytes prevents P75ntr-dependent motor neuron apoptosis. J Neurochem. (2006) 97:687–96. doi: 10.1111/j.1471-4159.2006.03742.x 16524372

[48] Alici D Bulbul F Virit O Unal A Altindag A Alpak G. Evaluation of oxidative metabolism and oxidative DNA damage in patients with obsessive–compulsive disorder. Psychiatry Clin. Neurosci. (2016) 70:109–15. doi: 10.1111/pcn.12362 26388322

[49] Mandal PK Saharan S Tripathi M Murari G. Brain glutathione levels--a Novel biomarker for mild cognitive impairment and Alzheimer's disease. Biol Psychiatry. (2015) 78:702–10. doi: 10.1016/j.biopsych.2015.04.005 26003861

[50] Monin A Baumann PS Griffa A Xin L Mekle R Fournier M. Glutathione deficit impairs myelin maturation: relevance for white matter integrity in schizophrenia patients. Mol Psychiatry. (2015) 20:827–38. doi: 10.1038/mp.2014.88 25155877

[51] Iskusnykh IY Zakharova AA Pathak D. Glutathione in brain disorders and aging. Molecules. (2022) 27:324. doi: 10.3390/molecules27010324, PMID: 35011559 PMC8746815

[52] Janáky R Ogita K Pasqualotto BA Bains JS Oja SS Yoneda Y. Glutathione and signal transduction in the mammalian Cns. J Neurochem. (1999) 73:889–902. doi: 10.1046/j.1471-4159.1999.0730889.x 10461878

[53] Jia Z Hallur S Zhu H Li Y Misra HP. Potent upregulation of glutathione and Nad(P)H:Quinone oxidoreductase 1 by alpha-lipoic acid in human neuroblastoma Sh-Sy5y cells: protection against Neurotoxicant-elicited cytotoxicity. Neurochem Res. (2008) 33:790–800. doi: 10.1007/s11064-007-9496-5 17940886

[54] Valenzuela A Aspillaga M Vial S Guerra R. Selectivity of Silymarin on the increase of the glutathione content in different tissues of the rat. Planta Med. (1989) 55:420–2. doi: 10.1055/s-2006-962056 2813578

[55] Bruno F Naselli F Brancato D Volpes S Cardinale PS Saccone S. Effects of Pterostilbene on the cell division cycle of a neuroblastoma cell line (2024) 16:4152. doi: 10.3390/nu16234152, PMC11644761 39683545

[56] Shih AY Johnson DA Wong G Kraft AD Jiang L Erb H. Coordinate regulation of glutathione biosynthesis and release by Nrf2-expressing glia potently protects neurons from oxidative stress (2003) 23:3394–406. doi: 10.1523/JNEUROSCI.23-08-03394.2003, PMC6742304 12716947

[57] Kimura Y Kimura H. Hydrogen sulfide protects neurons from oxidative stress. JTFj. (2004) 18:1165–7. doi: 10.1096/fj.04-1815fje 15155563

[58] Huang S-F Othman A Koshkin A Fischer S Fischer D Zamboni N. Astrocyte glutathione maintains endothelial barrier stability. Redox Biol. (2020) 34:101576. doi: 10.1016/j.redox.2020.101576 32502899 PMC7267730

[59] Song J Park J Oh Y Lee JE. Glutathione suppresses cerebral infarct volume and cell death after ischemic injury: involvement of Foxo3 inactivation and Bcl2 expression. Oxidative Med Cell Longev. (2015) 2015:426069:1–11. doi: 10.1155/2015/426069 PMC4334940 25722793

[60] MacKinley M Ford SD Jeon P Théberge J Palaniyappan L. Central oxidative stress and early vocational outcomes in first episode psychosis: A 7-tesla magnetic resonance spectroscopy study of glutathione. Schizophr Bull. (2022) 48:921–30. doi: 10.1093/schbul/sbac012 35307736 PMC9212125

[61] Kumar J Liddle EB Fernandes CC Palaniyappan L Hall EL Robson SE. Glutathione and glutamate in schizophrenia: A 7t Mrs study. Mol Psychiatry. (2020) 25:873–82. doi: 10.1038/s41380-018-0104-7 29934548 PMC7156342

[62] Gilbert HF. Thiol/disulfide exchange equilibria and disulfide bond stability. Methods Enzymol. (1995) 251:8–28. doi: 10.1016/0076-6879(95)51107-5 7651233

[63] Danbolt NC. Glutamate Uptake. Prog Neurobiol. (2001) 65:1–105. doi: 10.1016/s0301-0082(00)00067-8, PMID: 11369436

[64] Zhang C Yang Y Liang W Wang T Wang S Wang X. Neuroprotection by urate on the mutant Hsod1-related cellular and Drosophila models of amyotrophic lateral sclerosis: implication for Gsh synthesis via activating Akt/Gsk3β/Nrf2/Gclc pathways. Brain Res Bull. (2019) 146:287–301. doi: 10.1016/j.brainresbull.2019.01.019 30690059

[65] Griffith OW Meister A. Origin and turnover of mitochondrial glutathione. Proc Natl Acad Sci USA. (1985) 82:4668–72. doi: 10.1073/pnas.82.14.4668, PMID: 3860816 PMC390447

[66] Hargreaves KM Pardridge WM. Neutral amino acid transport at the human blood-brain barrier. J Biol Chem. (1988) 263:19392–7. doi: 10.1016/S0021-9258(19)77645-5 2848825

[67] Rappeneau V Blaker A Petro JR Yamamoto BK Shimamoto A. Disruption of the glutamate–glutamine cycle involving astrocytes in an animal model of depression for males and females. Front. Behav. Neurosci. (2016) 10:231. doi: 10.3389/fnbeh.2016.00231 28018190 PMC5147055

[68] Carletti B Banaj N Piras F Bossù P. Schizophrenia and glutathione: A challenging story (2023) 13:1526. doi: 10.3390/jpm13111526 PMC10672475 38003841

[69] Arruebarrena Di Palma A Perk EA Carboni ME García-Mata C Budak H Tör M. The Isothiocyanate Sulforaphane induces respiratory burst oxidase homologue D-dependent reactive oxygen species production and regulates expression of stress response genes. Plant Direct. (2022) 6:e437. doi: 10.1002/pld3.437, PMID: 36091879 PMC9448665

[70] Vairagar PR Sarkate AP Nirmal NP Sakhale BK. “New perspectives and role of phytochemicals in biofilm inhibition,” in Recent Frontiers of Phytochemicals. Elsevier (2023). p. 413–431.

[71] Ruhee RT Suzuki K. The integrative role of Sulforaphane in preventing inflammation, oxidative stress and fatigue: a review of a potential protective phytochemical. Antioxidants. (2020) 9:521. doi: 10.3390/antiox9060521 32545803 PMC7346151

[72] Abdel-Massih RM Debs E Othman L Attieh J Cabrerizo FM. Glucosinolates, a natural chemical arsenal: more to tell than the Myrosinase story. Front Microbiol. (2023) 14:1130208. doi: 10.3389/fmicb.2023.1130208 37089539 PMC10114928

[73] Zhou Q Chen B Wang X Wu L Yang Y Cheng X. Sulforaphane protects against rotenone-induced neurotoxicity in vivo: involvement of the Mtor, Nrf2 and autophagy pathways. Sci Rep. (2016) 6:32206. doi: 10.1038/srep32206, PMID: 27553905 PMC4995453

[74] Kim J. Pre-clinical neuroprotective evidences and plausible mechanisms of Sulforaphane in Alzheimer's disease. Int J Mol Sci. (2021) 22:2929. doi: 10.3390/ijms22062929 33805772 PMC7999245

[75] Yoo I-H Kim M-J Kim J Sung J-J Park ST Ahn S-W. The anti-inflammatory effect of Sulforaphane in mice with experimental autoimmune encephalomyelitis. J Korean Med Sci. (2019) 34:e197. doi: 10.3346/jkms.2019.34.e197 31327180 PMC6639507

[76] Nadeem A Ahmad SF Al-Harbi NO Attia SM Bakheet SA Ibrahim KE. Nrf2 activator, Sulforaphane ameliorates autism-like symptoms through suppression of Th17 related signaling and rectification of oxidant-antioxidant imbalance in periphery and brain of Btbr T+Tf/J mice. Behav Brain Res. (2019) 364:213–24. doi: 10.1016/j.bbr.2019.02.031 30790585

[77] Sharma P Kaushik P Jain S Sharma BM Awasthi R Kulkarni GT. Efficacy of Ulinastatin and Sulforaphane alone or in combination in rat model of Streptozotocin diabetes induced vascular dementia. Clin Psychopharm Neurosci. (2021) 19:470–89. doi: 10.9758/cpn.2021.19.3.470, PMID: 34294616 PMC8316668

[78] Yao W Zhang J-c Dong C Zhuang C Hirota S Inanaga K. Effects of Amycenone on serum levels of tumor necrosis factor-Α, Interleukin-10, and depression-like behavior in mice after lipopolysaccharide administration. Pharmacol Biochem Behav. (2015) 136:7–12. doi: 10.1016/j.pbb.2015.06.012, PMID: 26150007

[79] Pańczyszyn-Trzewik P Stachowicz K Misztak P Nowak G Sowa-Kućma M. Repeated Sulforaphane treatment reverses depressive-like behavior and exerts antioxidant effects in the olfactory bulbectomy model in mice (2024) 17:762. doi: 10.3390/ph17060762, PMC11206991 38931429

[80] Ghazizadeh-Hashemi F Bagheri S Ashraf-Ganjouei A Moradi K Shahmansouri N Mehrpooya M. Efficacy and safety of Sulforaphane for treatment of mild to moderate depression in patients with history of cardiac interventions: A randomized, double-blind, placebo-controlled clinical trial. Psychiatry Clin Neurosci. (2021) 75:250–5. doi: 10.1111/pcn.13276 34033171

[81] Shiina A Kanahara N Sasaki T Oda Y Hashimoto T Hasegawa T. An open study of Sulforaphane-rich broccoli sprout extract in patients with schizophrenia. Clin Psychopharm Neurosci. (2015) 13:62–7. doi: 10.9758/cpn.2015.13.1.62 PMC4423155 25912539

[82] Zhang R Miao Q-W Zhu C-X Zhao Y Liu L Yang J. Sulforaphane ameliorates neurobehavioral deficits and protects the brain from amyloid Β deposits and peroxidation in mice with Alzheimer-like lesions. Am J Alzheimer's Dis Other Dement. (2015) 30:183–91. doi: 10.1177/1533317514542645, PMID: 25024455 PMC10852928

[83] Bahn G Park JS Yun UJ Lee YJ Choi Y Park JS. Nrf2/are pathway negatively regulates Bace1 expression and ameliorates cognitive deficits in mouse Alzheimer's models. Proc Natl Acad Sci USA. (2019) 116:12516–23. doi: 10.1073/pnas.1819541116, PMID: 31164420 PMC6589670

[84] Masci A Mattioli R Costantino P Baima S Morelli G Punzi P. Neuroprotective effect of *Brassica Oleracea* sprouts crude juice in a cellular model of Alzheimer's disease. Oxidative Med Cell Longev. (2015) 2015:781938:1–17. doi: 10.1155/2015/781938 PMC4477226 26180595

[85] Wang W Wei C Quan M Li T Jia J. Sulforaphane reverses the amyloid-Β oligomers induced depressive-like behavior. J Alzheimer's Dis. (2020) 78:127–37. doi: 10.3233/JAD-200397, PMID: 32925042

[86] Zhang R Zhang J Fang L Li X Zhao Y Shi W. Neuroprotective effects of Sulforaphane on cholinergic neurons in mice with Alzheimer’s disease-like lesions. Int J Molec Sci. (2014) 15:14396–410. doi: 10.3390/ijms150814396 25196440 PMC4159858

[87] Ahmed SM Luo L Namani A Wang XJ Tang X. Nrf2 signaling pathway: pivotal roles in inflammation. Biochim Biophys Acta Mol basis Dis. (2017) 1863:585–97. doi: 10.1016/j.bbadis.2016.11.005 27825853

[88] Li B Cui W Liu J Li R Liu Q Xie XH. Sulforaphane ameliorates the development of experimental autoimmune encephalomyelitis by antagonizing oxidative stress and Th17-related inflammation in mice. Exp Neurol. (2013) 250:239–49. doi: 10.1016/j.expneurol.2013.10.002, PMID: 24120440

[89] Dash PK Zhao J Orsi SA Zhang M Moore AN. Sulforaphane improves cognitive function administered following traumatic brain injury. Neurosci Lett. (2009) 460:103–7. doi: 10.1016/j.neulet.2009.04.028, PMID: 19515491 PMC2700200

[90] Konwinski RR Haddad R Chun JA Klenow S Larson SC Haab BB. Oltipraz, 3h-1,2-Dithiole-3-Thione, and Sulforaphane induce overlapping and protective antioxidant responses in murine microglial cells. Toxicol Lett. (2004) 153:343–55. doi: 10.1016/j.toxlet.2004.06.006, PMID: 15454310

[91] Mao L Yang T Li X Lei X Sun Y Zhao Y. Protective effects of Sulforaphane in experimental vascular cognitive impairment: contribution of the Nrf2 pathway. J Cereb Blood Flow Metab. (2019) 39:352–66. doi: 10.1177/0271678x18764083 29533123 PMC6365596

[92] Zimmerman AW Singh K Connors SL Liu H Panjwani AA Lee L-C. Randomized controlled trial of Sulforaphane and metabolite discovery in children with autism Spectrum disorder. Mol Autism. (2021) 12:38. doi: 10.1186/s13229-021-00447-5 34034808 PMC8146218

[93] Hei G Smith RC Li R Ou J Song X Zheng Y. Sulforaphane effects on cognition and symptoms in first and early episode schizophrenia: A randomized double-blind trial. Schizoph Bull Open. (2022) 3:sgac024. doi: 10.1093/schizbullopen/sgac024 PMC11205988 39144775

[94] Abdull Razis AF Konsue N Ioannides C. Isothiocyanates and xenobiotic detoxification. Mol Nutr Food Res. (2018) 62:e1700916. doi: 10.1002/mnfr.201700916 29288567

[95] Steele ML Fuller S Patel M Kersaitis C Ooi L Münch G. Effect of Nrf2 activators on release of glutathione, Cysteinylglycine and homocysteine by human U373 Astroglial cells. Redox Biol. (2013) 1:441–5. doi: 10.1016/j.redox.2013.08.006 24191238 PMC3814960

[96] Ramos-Gomez M Kwak MK Dolan PM Itoh K Yamamoto M Talalay P. Sensitivity to carcinogenesis is increased and Chemoprotective efficacy of enzyme inducers is lost in Nrf2 transcription factor-deficient mice. Proc Natl Acad Sci USA. (2001) 98:3410–5. doi: 10.1073/pnas.051618798 11248092 PMC30667

[97] Deponte M. Glutathione catalysis and the reaction mechanisms of glutathione-dependent enzymes. Biochim Biophys Acta. (2013) 1830:3217–66. doi: 10.1016/j.bbagen.2012.09.018 23036594

[98] Zhang M An C Gao Y Leak RK Chen J Zhang F. Emerging roles of Nrf2 and phase ii antioxidant enzymes in neuroprotection. Prog Neurobiol. (2013) 100:30–47. doi: 10.1016/j.pneurobio.2012.09.003, PMID: 23025925 PMC3623606

[99] Cecatto A Calvete E Nienow A Costa R Mendonça H Pazzinato A. Culture Systems in the Production and Quality of strawberry cultivars. Acta Sci Agron. (2013) 35:471–8. doi: 10.4025/actasciagron.v35i4.16552

[100] Asimakopoulou A Panopoulos P Chasapis CT Coletta C Zhou Z Cirino G. Selectivity of commonly used pharmacological inhibitors for cystathionine Β synthase (Cbs) and cystathionine Γ Lyase (Cse). Br J Pharmacol. (2013) 169:922–32. doi: 10.1111/bph.12171 23488457 PMC3687671

[101] Patel S Fedinec AL Liu J Weiss MA Pourcyrous M Harsono M. H(2)S mediates the vasodilator effect of Endothelin-1 in the cerebral circulation. Am J Phys Heart Circ Phys. (2018) 315:H1759–64. doi: 10.1152/ajpheart.00451.2018 PMC6336977 30265150

[102] Benedict AL Mountney A Hurtado A Bryan KE Schnaar RL Dinkova-Kostova AT. Neuroprotective effects of Sulforaphane after contusive spinal cord injury. J Neurotr. (2012) 29:2576–86. doi: 10.1089/neu.2012.2474, PMID: 22853439 PMC3495118

[103] Kim J Lee S Choi BR Yang H Hwang Y Park JH. Sulforaphane epigenetically enhances neuronal Bdnf expression and Trkb signaling pathways. Mol Nutr Food Res. (2017) 61:2929. doi: 10.1002/mnfr.201600194 27735126

[104] Zhao F Zhang J Chang N. Epigenetic modification of Nrf2 by Sulforaphane increases the Antioxidative and anti-inflammatory capacity in a cellular model of Alzheimer's disease. Eur J Pharmacol. (2018) 824:1–10. doi: 10.1016/j.ejphar.2018.01.046, PMID: 29382536

[105] Jong CJ Sandal P Schaffer SW. The role of taurine in mitochondria health: more than just an antioxidant. Molecules (Basel, Switzerland). (2021) 26:4913. doi: 10.3390/molecules26164913 34443494 PMC8400259

[106] Stipanuk MH Ueki I. Dealing with methionine/homocysteine sulfur: cysteine metabolism to taurine and inorganic sulfur. J Inherit Metab Dis. (2011) 34:17–32. doi: 10.1007/s10545-009-9006-9, PMID: 20162368 PMC2901774

[107] Tramonti A Contestabile R Florio R Nardella C Barile A Di Salvo ML. Easy assay method for human cysteine Sulfinic acid decarboxylase. Life (Basel, Switzerland). (2021) 11:438. doi: 10.3390/life11050438 34068845 PMC8153620

[108] Roede JR Hansen JM Go YM Jones DP. Maneb and Paraquat-mediated neurotoxicity: involvement of Peroxiredoxin/Thioredoxin system. Toxicol Sci. (2011) 121:368–75. doi: 10.1093/toxsci/kfr058 21402726 PMC3098961

[109] Jangra A Gola P Singh J Gond P Ghosh S Rachamalla M. Emergence of taurine as a therapeutic agent for neurological disorders. Neural Regen Res. (2024) 19:62–8. doi: 10.4103/1673-5374.374139, PMID: 37488845 PMC10479846

[110] Caletti G Olguins DB Pedrollo EF Barros HM Gomez R. Antidepressant effect of taurine in diabetic rats. Amino Acids. (2012) 43:1525–33. doi: 10.1007/s00726-012-1226-x, PMID: 22302366

[111] Chen C Pung D Leong V Hebbar V Shen G Nair S. Induction of detoxifying enzymes by garlic organosulfur compounds through transcription factor Nrf2: effect of chemical structure and stress signals. Free Radic Biol Med. (2004) 37:1578–90. doi: 10.1016/j.freeradbiomed.2004.07.021, PMID: 15477009

[112] Song Y Cho J-H Kim H Eum Y-J Cheong EN Choi S. Association between taurine level in the Hippocampus and major depressive disorder in young women: A proton magnetic resonance spectroscopy study at 7t. Biol Psychiatry. (2024) 95:465–72. doi: 10.1016/j.biopsych.2023.08.025 37678539

[113] Kim HY Kim HV Yoon JH Kang BR Cho SM Lee S. Taurine in drinking water recovers learning and memory in the adult app/Ps1 mouse model of Alzheimer's disease. Sci Rep. (2014) 4:7467. doi: 10.1038/srep07467 25502280 PMC4264000

[114] Wang Q Fan W Cai Y Wu Q Mo L Huang Z. Protective effects of taurine in traumatic brain injury via mitochondria and cerebral blood flow. Amino Acids. (2016) 48:2169–77. doi: 10.1007/s00726-016-2244-x, PMID: 27156064

[115] Santa-María I Hernández F Moreno FJ Avila J. Taurine, an inducer for tau polymerization and a weak inhibitor for amyloid-Beta-peptide aggregation. Neurosci Lett. (2007) 429:91–4. doi: 10.1016/j.neulet.2007.09.068 17976912

[116] Abuirmeileh AN Abuhamdah SM Ashraf A Alzoubi KH. Protective effect of caffeine and/or taurine on the 6-Hydroxydopamine-induced rat model of Parkinson's disease: behavioral and neurochemical evidence. Restor Neurol Neurosci. (2021) 39:149–57. doi: 10.3233/rnn-201131, PMID: 33998560

[117] Che Y Hou L Sun F Zhang C Liu X Piao F. Taurine protects dopaminergic neurons in a mouse Parkinson's disease model through inhibition of microglial M1 polarization. Cell Death Dis. (2018) 9:435. doi: 10.1038/s41419-018-0468-2 29568078 PMC5864871

[118] Menzie J Prentice H Wu JY. Neuroprotective mechanisms of taurine against ischemic stroke. Brain Sci. (2013) 3:877–907. doi: 10.3390/brainsci3020877 24961429 PMC4061860

[119] Rak K Völker J Jürgens L Scherzad A Schendzielorz P Radeloff A. Neurotrophic effects of taurine on spiral ganglion neurons in vitro. Neuroreport. (2014) 25:1250–4. doi: 10.1097/wnr.0000000000000254, PMID: 25202928

[120] Singh P Gollapalli K Mangiola S Schranner D Yusuf MA Chamoli M. Taurine deficiency as a driver of aging. Science. (2023) 380:eabn9257. doi: 10.1126/science.abn9257 37289866 PMC10630957

[121] Wharton BA Morley R Isaacs EB Cole TJ Lucas A. Low plasma taurine and later neurodevelopment. Arch Dis Child Fetal Neonatal Ed. (2004) 89:F497–8. doi: 10.1136/adc.2003.048389 15499140 PMC1721794

[122] Abud GF De Carvalho FG Batitucci G Travieso SG Bueno Junior CR Barbosa Junior F. Taurine as a possible antiaging therapy: A controlled clinical trial on taurine antioxidant activity in women ages 55 to 70. Nutrition. (2022) 101:111706. doi: 10.1016/j.nut.2022.111706 35700594

[123] Zhu Y Wang R Fan Z Luo D Cai G Li X. Taurine alleviates chronic social defeat stress-induced depression by protecting cortical neurons from dendritic spine loss. Cell Mol Neurobiol. (2023) 43:827–40. doi: 10.1007/s10571-022-01218-3 35435537 PMC9958166

[124] Wu G-F Ren S Tang R-Y Xu C Zhou J-Q Lin S-M. Antidepressant effect of taurine in chronic unpredictable mild stress-induced depressive rats. Sci Rep. (2017) 7:4989. doi: 10.1038/s41598-017-05051-3 28694433 PMC5504064

[125] O'Donnell CP Allott KA Murphy BP Yuen HP Proffitt TM Papas A. Adjunctive taurine in first-episode psychosis: A phase 2, double-blind, randomized, placebo-controlled study. J Clin Psychiatry. (2016) 77:e1610–7. doi: 10.4088/JCP.15m10185, PMID: 27835719

[126] Ohsawa Y Hagiwara H Nishimatsu SI Hirakawa A Kamimura N Ohtsubo H. Taurine supplementation for prevention of stroke-like episodes in Melas: A multicentre, open-label, 52-week phase iii trial. J Neurol Neurosurg Psychiatry. (2019) 90:529–36. doi: 10.1136/jnnp-2018-317964, PMID: 29666206 PMC6581075

[127] Baliou S Kyriakopoulos AM Goulielmaki M Panayiotidis MI Spandidos DA Zoumpourlis V. Significance of taurine transporter (taut) in homeostasis and its layers of regulation. Molec Med Rep. (2020) 22:2163–73. doi: 10.3892/mmr.2020.11321, PMID: 32705197 PMC7411481

[128] Paula-Lima AC De Felice FG Brito-Moreira J Ferreira ST. Activation of Gaba(a) receptors by taurine and Muscimol blocks the neurotoxicity of Beta-amyloid in rat hippocampal and cortical neurons. Neuropharmacology. (2005) 49:1140–8. doi: 10.1016/j.neuropharm.2005.06.015 16150468

[129] Kocis P Tolar M Yu J Sinko W Ray S Blennow K. Elucidating the Aβ42 anti-aggregation mechanism of action of Tramiprosate in Alzheimer's disease: integrating molecular analytical methods, pharmacokinetic and clinical data. CNS Drugs. (2017) 31:495–509. doi: 10.1007/s40263-017-0434-z, PMID: 28435985 PMC5488121

[130] Jakaria M Azam S Haque ME Jo SH Uddin MS Kim IS. Taurine and its analogs in neurological disorders: focus on therapeutic potential and molecular mechanisms. Redox Biol. (2019) 24:101223. doi: 10.1016/j.redox.2019.101223 31141786 PMC6536745

[131] Sha D Wei J Jin H Wu H Osterhaus GL Wu J-Y. Effect of taurine on regulation of Gaba and acetylcholine biosynthesis In: Lombardini JB Schaffer SW Azuma J, editors. Taurine 5: Beginning the 21st century. Boston, MA: Springer US (2003). 499–505. 10.1007/978-1-4615-0077-3_60 12908636

[132] Ahmed S Ma N Kawanokuchi J Matsuoka K Oikawa S Kobayashi H. Taurine reduces microglia activation in the brain of aged senescence-accelerated mice by increasing the level of Trem2. Sci Rep. (2024) 14:7427. doi: 10.1038/s41598-024-57973-4, PMID: 38548872 PMC10978912

[133] Schaffer S Kim HW. Effects and mechanisms of taurine as a therapeutic agent. Biomol Ther. (2018) 26:225–41. doi: 10.4062/biomolther.2017.251 PMC5933890 29631391

[134] Cunha L Grenha A. Sulfated seaweed polysaccharides as multifunctional materials in drug delivery applications. Mar Drugs. (2016) 14:42. doi: 10.3390/md14030042 26927134 PMC4820297

[135] Knutsen S Myslabodski D Larsen B Usov A. A Modified System of Nomenclature for Red Algal Galactans. Botanica Marina - BOT MAR. (1994) 37:163–70. doi: 10.1515/botm.1994.37.2.163

[136] Usov AI. Polysaccharides of the Red Algae. Adv Carbohydr Chem Biochem. (2011) 65:115–217. doi: 10.1016/B978-0-12-385520-6.00004-2 21763512

[137] Percival EGV Ross AG. 145. Fucoidin. Part I. The isolation and purification of Fucoidin from Brown seaweeds. J Chem Soc. (1950) 717–20. doi: 10.1039/JR9500000717

[138] Leung MY Liu C Koon JC Fung KP. Polysaccharide biological response modifiers. Immunol Lett. (2006) 105:101–14. doi: 10.1016/j.imlet.2006.01.009, PMID: 16554097

[139] Kidgell JT Magnusson M de Nys R Glasson CRK. Ulvan: A systematic review of extraction, composition and function. Algal Res. (2019) 39:101422. doi: 10.1016/j.algal.2019.101422

[140] Robic A Bertrand D Sassi J-F Lerat Y Lahaye MJ. Determination of the chemical composition of ulvan, a cell wall polysaccharide from Ulva spp. (Ulvales, Chlorophyta) by FT-IR and chemometrics. J Appl Phycol. (2009) 21:451–6. doi: 10.1007/s10811-008-9390-9, PMID: 40082651

[141] Zhou Y Duan Y Huang S Zhou X Zhou L Hu T. Polysaccharides from *Lycium Barbarum* ameliorate amyloid pathology and cognitive functions in app/Ps1 transgenic mice. Int J Biol Macromol. (2020) 144:1004–12. doi: 10.1016/j.ijbiomac.2019.09.177, PMID: 31715236

[142] Xing M Li G Liu Y Yang L Zhang Y Zhang Y. Fucoidan from *Fucus Vesiculosus* prevents the loss of dopaminergic neurons by alleviating mitochondrial dysfunction through targeting Atp5f1a. Carbohydr Polym. (2023) 303:120470. doi: 10.1016/j.carbpol.2022.120470, PMID: 36657849

[143] Li M Sun X Li Q Li Y Luo C Huang H. Fucoidan exerts antidepressant-like effects in mice via regulating the stability of surface Ampars. Biochem Biophys Res Commun. (2020) 521:318–25. doi: 10.1016/j.bbrc.2019.10.043, PMID: 31668812

[144] Lee B Shim I Lee H Hahm DH. Fucoidan prevents depression-like behavior in rats exposed to repeated restraint stress. J Nat Med. (2013) 67:534–44. doi: 10.1007/s11418-012-0712-5 23090005

[145] Park HY Han MH Park C Jin CY Kim GY Choi IW. Anti-inflammatory effects of Fucoidan through inhibition of Nf-Κb, Mapk and Akt activation in lipopolysaccharide-induced Bv2 microglia cells. Food Chem Toxicol. (2011) 49:1745–52. doi: 10.1016/j.fct.2011.04.020 21570441

[146] Olasehinde TA Mabinya LV Olaniran AO Okoh AI. Chemical characterization, antioxidant properties, cholinesterase inhibitory and anti-Amyloidogenic activities of sulfated polysaccharides from some seaweeds. Bioact Carbohydr Diet Fibre. (2019) 18:100182. doi: 10.1016/j.bcdf.2019.100182

[147] Han YS Lee JH Lee SH. Fucoidan suppresses mitochondrial dysfunction and cell death against 1-Methyl-4-Phenylpyridinum-induced neuronal cytotoxicity via regulation of Pgc-1α expression. Mar Drugs. (2019) 17:518. doi: 10.3390/md17090518 31480724 PMC6780744

[148] Nambi P Sathyamoorthy Y Kaliyappan K Radhakrishnan R. Fucoidan (a sulfated polysaccharide) and Cerebroprotein in combination alleviate the Neuroinflammation-mediated neural damage and functional deficits in the focal cerebral ischemia model of rat. Neuroscience. (2023) 524:52–64. doi: 10.1016/j.neuroscience.2023.05.003, PMID: 37182836

[149] Allaert FA Demais H Collén PN. A randomized controlled double-blind clinical trial comparing versus placebo the effect of an edible algal extract (*Ulva Lactuca*) on the component of depression in healthy volunteers with anhedonia. BMC Psychiatry. (2018) 18:215. doi: 10.1186/s12888-018-1784-x, PMID: 29954354 PMC6027788

[150] Subermaniam K Teoh SL Yow YY Tang YQ Lim LW Wong KH. Marine algae as emerging therapeutic alternatives for depression: A review. Iran J Basic Med Sci. (2021) 24:997–1013. doi: 10.22038/ijbms.2021.54800.12291 34804417 PMC8591755

[151] Patel S. Therapeutic importance of sulfated polysaccharides from seaweeds: updating the recent findings: 3. Biotech. (2012) 2:171–85. doi: 10.1007/s13205-012-0061-9

[152] Lomartire S Gonçalves AM. Marine macroalgae polyphenols as potential neuroprotective antioxidants in neurodegenerative diseases. Marine Drugs. (2023) 21:261. doi: 10.3390/md21050261, PMID: 37233455 PMC10221822

[153] Perron NR Brumaghim JL. Biophysics. A review of the antioxidant mechanisms of polyphenol compounds related to Iron binding. Cell Biochem Biophy. (2009) 53:75–100. doi: 10.1007/s12013-009-9043-x, PMID: 19184542

[154] Anisha GS Padmakumari S Patel AK Pandey A Singhania RR. Fucoidan from marine macroalgae: biological actions and applications in regenerative medicine, drug delivery systems and food industry. Bioengineering (Basel, Switzerland). (2022) 9:472. doi: 10.3390/bioengineering9090472, PMID: 36135017 PMC9495336

[155] Dhahri M Alghrably M Mohammed HA Badshah SL Noreen N Mouffouk F. Natural polysaccharides as preventive and therapeutic horizon for neurodegenerative diseases. Pharmaceutics. (2021) 14:1. doi: 10.3390/pharmaceutics14010001, PMID: 35056897 PMC8777698

[156] Mahomoodally MF Nabee N Baureek N. “Organosulfur compounds (allyl sulfide, indoles),” in Antioxidants Effects in Health. Elsevier (2022). p. 417–426. doi: 10.1016/B978-0-12-819096-8.00070-7

[157] Omar SH Al-Wabel NA. Organosulfur compounds and possible mechanism of garlic in Cancer. Saudi Pharma J. (2010) 18:51–8. doi: 10.1016/j.jsps.2009.12.007, PMID: 23960721 PMC3731019

[158] Amagase H Petesch BL Matsuura H Kasuga S Itakura Y. Intake of garlic and its bioactive components. J Nutr. (2001) 131:955S–62S. doi: 10.1093/jn/131.3.955S 11238796

[159] Zhang H Shang C Tian Z Amin HK Kassab RB Abdel Moneim AE. Diallyl disulfide suppresses inflammatory and oxidative machineries following carrageenan injection-induced paw edema in mice. Mediat Inflamm. (2020) 2020:1–11. doi: 10.1155/2020/8508906 PMC7180418 32377166

[160] Wei X Ma Y Li F He H Huang H Huang C. Acute Diallyl disulfide administration prevents and reveres lipopolysaccharide-induced depression-like behaviors in mice via regulating Neuroinflammation and Oxido-Nitrosative stress. Inflammation. (2021) 44:1381–95. doi: 10.1007/s10753-021-01423-0 33511484

[161] Guo Y Zhang K Wang Q Li Z Yin Y Xu Q. Neuroprotective effects of Diallyl Trisulfide in Sod1-G93a transgenic mouse model of amyotrophic lateral sclerosis. Brain Res. (2011) 1374:110–5. doi: 10.1016/j.brainres.2010.12.014 21147075

[162] Ray B Chauhan NB Lahiri DK. Oxidative insults to neurons and synapse are prevented by aged garlic extract and S-allyl-L-cysteine treatment in the neuronal culture and app-Tg mouse model. J Neurochem. (2011) 117:388–402. doi: 10.1111/j.1471-4159.2010.07145.x, PMID: 21166677 PMC3391571

[163] Rahmani G Farajdokht F Mohaddes G Babri S Ebrahimi V Ebrahimi H. Garlic (*Allium Sativum*) improves anxiety- and depressive-related behaviors and brain oxidative stress in diabetic rats. Arch Physiol Biochem. (2020) 126:95–100. doi: 10.1080/13813455.2018.1494746 30169970

[164] Gao W Wang W Liu G Zhang J Yang J Deng Z. Allicin attenuated chronic social defeat stress induced depressive-like behaviors through suppression of Nlrp3 Inflammasome. Metab Brain Dis. (2019) 34:319–29. doi: 10.1007/s11011-018-0342-z, PMID: 30515710

[165] Chai JX Li HH Wang YY Chai Q He WX Zhou YM. Effect of Diallyl disulfide on learning and memory abilities and hippocampal synapses in mouse models of Alzheimer's disease. J South Med Univ. (2016) 36:1417–22. PMID: 27777209

[166] Carlos S-I Ricardo AS Ana LC-G Perla DM. Nrf2 activation, an innovative therapeutic alternative in cerebral ischemia In: Maurizio B, editor. Advances in the preclinical study of ischemic stroke. Rijeka: IntechOpen (2012). 15.

[167] Zhuang F Shi X Qiao S Liu B Wang Z Huo H. Allicin promotes functional recovery in ischemic stroke via glutathione Peroxidase-1 activation of Src-Akt-Erk. Cell Death Disc. (2023) 9:335. doi: 10.1038/s41420-023-01633-5, PMID: 37673878 PMC10482956

[168] Ruiz-Sánchez E Pedraza-Chaverri J Medina-Campos ON Maldonado PD Rojas P. S-allyl cysteine, a garlic compound, produces an antidepressant-like effect and exhibits antioxidant properties in mice. Brain Sci. (2020) 10:592. doi: 10.3390/brainsci10090592 32859119 PMC7564461

[169] Gong P Hu B Cederbaum AI. Diallyl sulfide induces Heme Oxygenase-1 through Mapk pathway. Arch Biochem Biophys. (2004) 432:252–60. doi: 10.1016/j.abb.2004.09.024, PMID: 15542064

[170] Wang G Yang Y Wang C Huang J Wang X Liu Y. Exploring the role and mechanisms of Diallyl Trisulfide and Diallyl disulfide in chronic constriction-induced neuropathic pain in rats. Korean J Pain. (2020) 33:216–25. doi: 10.3344/kjp.2020.33.3.216, PMID: 32606266 PMC7336342

[171] Buendia I Michalska P Navarro E Gameiro I Egea J León R. Nrf2-are pathway: An emerging target against oxidative stress and Neuroinflammation in neurodegenerative diseases. Pharmacol Ther. (2016) 157:84–104. doi: 10.1016/j.pharmthera.2015.11.003 26617217

[172] Neumann M Sampathu DM Kwong LK Truax AC Micsenyi MC Chou TT. Ubiquitinated Tdp-43 in frontotemporal lobar degeneration and amyotrophic lateral sclerosis. Science (New York, NY). (2006) 314:130–3. doi: 10.1126/science.1134108 17023659

[173] Liu C Leng B Li Y Jiang H Duan W Guo Y. Diallyl Trisulfide protects motor neurons from the neurotoxic protein Tdp-43 via activating lysosomal degradation and the antioxidant response. Neurochem Res. (2018) 43:2304–12. doi: 10.1007/s11064-018-2651-3 30317421

[174] Huang Q Aluise CD Joshi G Sultana R St Clair DK Markesbery WR. Potential in vivo amelioration by N-acetyl-L-cysteine of oxidative stress in brain in human double mutant app/Ps-1 Knock-in mice: toward therapeutic modulation of mild cognitive impairment. J Neurosci Res. (2010) 88:2618–29. doi: 10.1002/jnr.22422 20648652

[175] Rehman T Shabbir MA Inam-Ur-Raheem M Manzoor MF Ahmad N Liu ZW. Cysteine and homocysteine as biomarker of various diseases. Food Sci Nutr. (2020) 8:4696–707. doi: 10.1002/fsn3.1818 32994931 PMC7500767

[176] Semenza ER Harraz MM Abramson E Malla AP Vasavda C Gadalla MM. D-cysteine is an endogenous regulator of neural progenitor cell dynamics in the mammalian brain. Proc Natl Acad Sci USA. (2021) 118:e2110610118. doi: 10.1073/pnas.2110610118 34556581 PMC8488618

[177] Sbodio JI Snyder SH Paul BD. Golgi stress response reprograms cysteine metabolism to confer Cytoprotection in Huntington's disease. Proc Natl Acad Sci USA. (2018) 115:780–5. doi: 10.1073/pnas.1717877115 29317536 PMC5789946

[178] Jalgaonkar S Gajbhiye S Sayyed M Tripathi R Khatri N Parmar U. S-Adenosyl methionine improves motor co-ordination with reduced oxidative stress, dopaminergic neuronal loss, and DNA methylation in the brain striatum of 6-Hydroxydopamine-induced neurodegeneration in rats. Anatom Rec (Hoboken, NJ). (2023) 306:820–30. doi: 10.1002/ar.24948 35476228

[179] ElBaz FM Zaki MM Youssef AM ElDorry GF DYJE E. Study of plasma amino acid levels in children with autism: an Egyptian sample. Egyp J Med Hum Gen. (2014) 15:181–6. doi: 10.1016/j.ejmhg.2014.02.002, PMID: 40088920

[180] Khan M Sekhon B Jatana M Giri S Gilg AG Sekhon C. Administration of N-acetylcysteine after focal cerebral ischemia protects brain and reduces inflammation in a rat model of experimental stroke. J Neurosci Res. (2004) 76:519–27. doi: 10.1002/jnr.20087 15114624

[181] Monti DA Zabrecky G Kremens D Liang T-W Wintering NA Cai J. N-acetyl cysteine may support dopamine neurons in Parkinson's disease: preliminary clinical and cell line data (2016) 11:e0157602. doi: 10.1371/journal.pone.0157602, PMC4911055 27309537

[182] Santos P Herrmann AP Benvenutti R Noetzold G Giongo F Gama CS. Anxiolytic properties of N-acetylcysteine in mice. Behav Brain Res. (2017) 317:461–9. doi: 10.1016/j.bbr.2016.10.010 27725170

[183] Magalhães PV Dean OM Bush AI Copolov DL Malhi GS Kohlmann K. A preliminary investigation on the efficacy of N-acetyl cysteine for mania or hypomania. Aust N Z J Psychiatry. (2013) 47:564–8. doi: 10.1177/0004867413481631, PMID: 23493756

[184] Rapado-Castro M Berk M Venugopal K Bush AI Dodd S Dean OM. Towards stage specific treatments: effects of duration of illness on therapeutic response to adjunctive treatment with N-acetyl cysteine in schizophrenia. Prog Neuro-Psychopharmacol Biol Psychiatry. (2015) 57:69–75. doi: 10.1016/j.pnpbp.2014.10.002 25315856

[185] Conus P Seidman LJ Fournier M Xin L Cleusix M Baumann PS. N-acetylcysteine in a double-blind randomized placebo-controlled trial: toward biomarker-guided treatment in early psychosis. Schizophr Bull. (2017) 44:317–27. doi: 10.1093/schbul/sbx093 PMC5815074 29462456

[186] Sepehrmanesh Z Heidary M Akasheh N Akbari H Heidary M. Therapeutic effect of adjunctive N-acetyl cysteine (Nac) on symptoms of chronic schizophrenia: A double-blind, randomized clinical trial. Prog Neuro-Psychopharmacol Biol Psychiatry. (2018) 82:289–96. doi: 10.1016/j.pnpbp.2017.11.001, PMID: 29126981

[187] Xu Y Yang Y Shi Y Li B Xie Y Le G. Dietary methionine supplementation improves cognitive dysfunction associated with Transsulfuration pathway upregulation in subacute aging mice. NPJ Sci Food. (2024) 8:104. doi: 10.1038/s41538-024-00348-w, PMID: 39702349 PMC11659567

[188] Holper S Watson R Churilov L Yates P Lim YY Barnham KJ. Protocol of a phase ii randomized, multi-center, double-blind, placebo-controlled trial of S-Adenosyl methionine in participants with mild cognitive impairment or dementia due to Alzheimer's disease. J Prev Alzheimers Dis. (2023) 10:800–9. doi: 10.14283/jpad.2023.55 37874102 PMC10186290

[189] Salmaggi P Bressa GM Nicchia G Coniglio M La Greca P Le Grazie C. Double-blind, placebo-controlled study of S-Adenosyl-L-methionine in depressed postmenopausal women. Psychother Psychosom. (1993) 59:34–40. doi: 10.1159/000288642, PMID: 8441793

[190] Fava M Giannelli A Rapisarda V Patralia A Guaraldi GP. Rapidity of onset of the antidepressant effect of parenteral S-Adenosyl-L-methionine. Psychiatry Res. (1995) 56:295–7. doi: 10.1016/0165-1781(95)02656-h 7568552

[191] Fu AL Dong ZH Sun MJ. Protective effect of N-acetyl-L-cysteine on amyloid Beta-peptide-induced learning and memory deficits in mice. Brain Res. (2006) 1109:201–6. doi: 10.1016/j.brainres.2006.06.042, PMID: 16872586

[192] Monti D Zabrecky G Kremens D Liang T-W Wintering N Bazzan A. N-acetyl cysteine is associated with dopaminergic improvement in Parkinson's disease. Clin Pharma Therap. (2019) 106:884–90. doi: 10.1002/cpt.1548, PMID: 31206613

[193] Knuckey NW Palm D Primiano M Epstein MH Johanson CE. N-acetylcysteine enhances hippocampal neuronal survival after transient forebrain ischemia in rats. Stroke. (1995) 26:305–11. doi: 10.1161/01.str.26.2.305 7831704

[194] Louapre C Perlbarg V García-Lorenzo D Urbanski M Benali H Assouad R. Brain networks disconnection in early multiple sclerosis cognitive deficits: An Anatomofunctional study. Hum Brain Mapp. (2014) 35:4706–17. doi: 10.1002/hbm.22505, PMID: 24687771 PMC6869493

[195] Mishra S Kumar G Chhabra A Sethy NK Jain N Meena RN. Cysteine becomes conditionally essential during hypobaric hypoxia and regulates adaptive neuro-physiological responses through Cbs/H2s pathway. Biochim Biophys Acta (BBA)—Mol Basis Dis. (2020) 1866:165769. doi: 10.1016/j.bbadis.2020.165769 32184133

[196] Shahrampour S Heholt J Wang A Vedaei F Mohamed FB Alizadeh M. N-acetyl cysteine administration affects cerebral blood flow as measured by arterial spin labeling Mri in patients with multiple sclerosis. Heliyon. (2021) 7:e07615. doi: 10.1016/j.heliyon.2021.e07615, PMID: 34377857 PMC8327674

[197] Lail H Mabb AM Parent MB Pinheiro F Wanders D. Effects of dietary methionine restriction on cognition in mice. Nutrients. (2023) 15:4950. doi: 10.3390/nu15234950 38068808 PMC10707861

[198] Kumar G Chhabra A Mishra S Kalam H Kumar D Meena R. H2s regulates hypobaric hypoxia-induced early Glio-vascular dysfunction and neuro-pathophysiological effects. EBioMedicine. (2016) 6:171–89. doi: 10.1016/j.ebiom.2016.03.002, PMID: 27211559 PMC4856789

[199] Guo C Liang F Shah Masood W Yan X. Hydrogen sulfide protected gastric epithelial cell from ischemia/reperfusion injury by Keap1 S-Sulfhydration, Mapk dependent anti-apoptosis and Nf-Κb dependent anti-inflammation pathway. Eur J Pharmacol. (2014) 725:70–8. doi: 10.1016/j.ejphar.2014.01.009 24444438

[200] Hourihan JM Kenna JG Hayes JD. The Gasotransmitter hydrogen sulfide induces Nrf2-target genes by inactivating the Keap1 ubiquitin ligase substrate adaptor through formation of a disulfide bond between Cys-226 and Cys-613. Antioxid Redox Signal. (2013) 19:465–81. doi: 10.1089/ars.2012.4944 23145493

[201] Yang G Zhao K Ju Y Mani S Cao Q Puukila S. Hydrogen sulfide protects against cellular senescence via S-Sulfhydration of Keap1 and activation of Nrf2. Antioxid Redox Signal. (2013) 18:1906–19. doi: 10.1089/ars.2012.4645, PMID: 23176571

[202] Dringen R Pfeiffer B Hamprecht B. Synthesis of the antioxidant glutathione in neurons: supply by astrocytes of Cysgly as precursor for neuronal glutathione. J Neurosci. (1999) 19:562–9. doi: 10.1523/jneurosci.19-02-00562.1999, PMID: 9880576 PMC6782200

[203] Qin S Colin C Hinners I Gervais A Cheret C Mallat M. System xc- and apolipoprotein E expressed by microglia have opposite effects on the neurotoxicity of amyloid-Beta peptide 1-40. J Neurosci. (2006) 26:3345–56. doi: 10.1523/jneurosci.5186-05.2006, PMID: 16554485 PMC6674113

[204] Mesci P Zaïdi S Lobsiger CS Millecamps S Escartin C Seilhean D. System xc− is a mediator of microglial function and its deletion slows symptoms in amyotrophic lateral sclerosis mice. Brain. (2014) 138:53–68. doi: 10.1093/brain/awu312 25384799 PMC4441079

[205] Latimer MN Freij KW Cleveland BM Biga PR. Physiological and molecular mechanisms of methionine restriction. Front Endocrinol. (2018) 9:217. doi: 10.3389/fendo.2018.00217, PMID: 29780356 PMC5945823

[206] Hooshmand B Refsum H Smith AD Kalpouzos G Mangialasche F von Arnim CAF. Association of Methionine to homocysteine status with brain magnetic resonance imaging measures and risk of dementia. JAMA Psychiatry. (2019) 76:1198–205. doi: 10.1001/jamapsychiatry.2019.1694 31339527 PMC6659152

[207] Pi T Liu B Shi J. Abnormal homocysteine metabolism: An insight of Alzheimer's disease from DNA methylation. Behav Neurol. (2020) 2020:1–11. doi: 10.1155/2020/8438602 PMC7495165 32963633

[208] Smith LN. What's right and wrong in preclinical science: A matter of principled investigation. Front Behav Neurosci. (2022) 16:805661. doi: 10.3389/fnbeh.2022.805661, PMID: 35355924 PMC8959833

[209] Kagan BL Sultzer DL Rosenlicht N Gerner RH. Oral S-Adenosylmethionine in depression: A randomized, double-blind, placebo-controlled trial. Am J Psychiatry. (1990) 147:591–5. doi: 10.1176/ajp.147.5.591 2183633

